# Consequences of cognitive offloading: Boosting performance but diminishing memory

**DOI:** 10.1177/17470218211008060

**Published:** 2021-04-04

**Authors:** Sandra Grinschgl, Frank Papenmeier, Hauke S Meyerhoff

**Affiliations:** 1 Department of Psychology, University of Tübingen, Tübingen, Germany; 2 Department of Psychology, University of Graz, Graz, Austria; 3 Leibniz-Institut für Wissensmedien, Tübingen, Germany

**Keywords:** Cognitive offloading, working memory, external memory, technology

## Abstract

Modern technical tools such as tablets allow for the temporal externalisation of working memory processes (i.e., cognitive offloading). Although such externalisations support immediate performance on different tasks, little is known about potential long-term consequences of offloading behaviour. In the current set of experiments, we studied the relationship between cognitive offloading and subsequent memory for the offloaded information as well as the interplay of this relationship with the goal to acquire new memory representations. Our participants solved the Pattern Copy Task, in which we manipulated the costs of cognitive offloading and the awareness of a subsequent memory test. In Experiment 1 (*N* = 172), we showed that increasing the costs for offloading induces reduced offloading behaviour. This reduction in offloading came along with lower immediate task performance but more accurate memory in an unexpected test. In Experiment 2 (*N* = 172), we confirmed these findings and observed that offloading behaviour remained detrimental for subsequent memory performance when participants were aware of the upcoming memory test. Interestingly, Experiment 3 (*N* = 172) showed that cognitive offloading is not detrimental for long-term memory formation under all circumstances. Those participants who were forced to offload maximally but were aware of the memory test could almost completely counteract the negative impact of offloading on memory. Our experiments highlight the importance of the explicit goal to acquire new memory representations when relying on technical tools as offloading did have detrimental effects on memory without such a goal.

Already 2.5 million years ago, ancestors of *Homo sapiens* used tools to improve performance on particular tasks (e.g., using stones for butchery; Ambrose, 2001). Since then, tools have been developed further and modern technologies not only support physical actions but also allow for the externalisation of cognitive processes (see Osiurak et al., 2018). Tablets and smartphones are examples of such modern technical tools, and they have become ubiquitous in everyday life (Greenhow et al., 2009). Although most of the tools support immediate performance, such as performing a task faster and/or with fewer errors, potential negative effects following their extensive use have been discussed for cognitive tools (e.g., impaired scene recognition after using navigation systems; Fenech et al., 2010). In the present set of experiments, we studied memory as one of the most fundamental cognitive processes. Specifically, we investigated a trade-off between immediate performance and subsequent mental representations due to cognitive offloading.

## Cognitive offloading

Using tools for externalising cognitive processes is typically referred to as *cognitive offloading*, which can be defined as “the use of physical action to alter the information processing requirements of a task so as to reduce cognitive demand” (Risko & Gilbert, 2016). With regard to working memory, the act of cognitive offloading releases resources which otherwise would be necessary to actively hold information in short-term representations. Instead, the corresponding information is externalised into technical tools such as mobile touch devices (Wilson, 2002). Whether humans tend to offload cognitive processes such as memory often depends on cost–benefit evaluations of internal processing versus externalisation. Raising the costs of externalisations (e.g., by adding additional physical or temporal demands) increases the use of internal strategies such as memory-based processing, whereas lowering the costs of externalisations increases the use of technical tools (e.g., Cary & Carlson, 2001; Gray & Fu, 2004; Gray et al., 2006; Morgan & Patrick, 2013; O’Hara & Payne, 1998; Schönpflug, 1986). However, not all decisions to offload cognitive processes follow cost–benefit considerations of different strategies but may also be influenced by the current goals of the observers (Weis & Wiese, 2019) as well as their tendency to avoid demanding cognitive actions (Ballard et al., 1997; Kool et al., 2010; Sachdeva & Gilbert, 2020).

A typical working memory task used to study the externalisation of memory is the Pattern Copy Task (called Blocks World Task in previous work; Ballard et al., 1992, 1995). In this task, the participants replicate a colour pattern displayed in a model window on one side of the screen in an empty workspace window on the other side of the screen. In most versions of the task, the model window and workspace window are not visible at the same time, but instead one window is covered by a grey mask whenever the other one is uncovered (e.g., Fu & Gray, 2000; Gray et al., 2006). This design allows participants to decide whether they prefer to rely on internal memorisation (indicated by fewer switches between the two windows) or to rely on externalisations of the memory processes (indicated by additional physical efforts for more switches between the two windows). The participants’ decision between internal memorisation and externalisation within this task depends on subjective cost–benefit considerations. With regard to these considerations, the work of Fu and Gray (2000; see also Gray et al., 2006; Grinschgl, Meyerhoff, & Papenmeier, 2020; Patrick et al., 2015; Waldron et al., 2011) has shown that increasing access costs by adding temporal delays for each inspection of the model window results in a shift from offloading strategies to more memory-based strategies. Thus, higher temporal costs reduce offloading behaviour. In the present research, we use this robust and consistent finding to experimentally investigate offloading behaviour and its immediate impact on performance as well as subsequent memory.

## Consequences of cognitive offloading

Conceptually, externalisations of cognitive processes into a technical tool could be considered an extended mind as externalisations spread cognitive processes beyond the boundaries of the individual mind (Clark & Chalmers, 1998). Empirically, such externalisations have been shown to improve problem-solving accuracy as well as speed (Kirsh & Maglio, 1994). Such beneficial effects on immediate task performance in terms of speed and/or accuracy have been observed across a large variety of tasks (e.g., arithmetic tasks, see Carlson et al., 2007; Goldin-Meadow et al., 2001; or reading, see Risko et al., 2014). Besides these described effects *with* technology (i.e., performing beyond internal cognitive constraints; Salomon, 1990), little is known about the effects *of* technology (i.e., the cognitive consequences of interactions with technology; Moritz et al., 2020; Salomon, 1990; Salomon & Perkins, 2005).

On one hand, offloading irrelevant information into technical tools improves cognitive performance for subsequent, unrelated tasks (Runge et al., 2019) as well as memory for unrelated information (Storm & Stone, 2015). On the other hand, however, a common concern states that frequent externalisation of internal cognitive processes leads to an impoverishment of the corresponding internal abilities. This concern has received empirical support from findings on spatial memory (e.g., Fenech et al., 2010; Gardony et al., 2015), problem solving (Moritz et al., 2018, 2020; O’Hara & Payne, 1998; Van Nimwegen & Van Oostendorp, 2009), as well as the recall of information (Eskritt & Ma, 2014; Kelly & Risko, 2019; Pyke & LeFevre, 2011; Sparrow et al., 2011). For instance, O’Hara and Payne (1998) observed that problem solving was less successful in a transfer phase following an increasing amount of interactions with a technical tool relative to using internal mental processes. Moreover, Kelly and Risko (2019) recently studied how relying on external representations affects memory accuracy for the offloaded information. Despite identical stimulus encoding, the participants of this study remembered word lists less accurately when they thought they would have access to external representations than when they thought they would have to rely on their internal memory.

Critically, in these lines of research, the participants typically could not choose how to perform the task while encoding task-relevant information (e.g., Eskritt & Ma, 2014; Kelly & Risko, 2019; Sparrow et al., 2011). This is different for the Pattern Copy Task, in which the participants can freely adapt their offloading behaviour (whereby at least some offloading is necessary to solve the task) and thus choose their preferred strategy. Nevertheless, research exploring the Pattern Copy Task points towards a similar trade-off between positive and negative effects of cognitive offloading (Morgan et al., 2009, 2013; Waldron et al., 2007). While an increasing amount of cognitive offloading (e.g., in conditions with low temporal costs relative to conditions with high temporal costs) accelerates task processing (Morgan et al., 2009; Waldron et al., 2007), it subsequently diminishes recall performance for visuospatial information (Morgan et al., 2009, 2013; Waldron et al., 2007). Furthermore, an increased amount of offloading was harmful for resumptions (i.e., continuing to rebuild the colour pattern from one’s memory) after task interruptions (Morgan et al., 2009, 2013). However, the reported studies tested the memory for the offloaded information immediately after offloading (Morgan et al., 2009, 2013; Waldron et al., 2007). The memory tests in these studies might therefore not have exceeded working memory maintenance. It still remains unclear whether the detrimental effects of offloading also persist at longer time intervals, thus affecting long-term memory. This is especially important, as in real-life situations individuals often offload information to access this information at a later stage (i.e., writing a shopping list or using a calendar). These considerations pose the research question whether cognitive offloading would also be harmful for long-term memory acquisition.

With regard to long-term memory acquisition, the awareness of the relevance of the offloaded information for subsequent testing might alter offloading behaviour itself as well as potential consequences. This is because being aware of a subsequent test should induce the goal to foster learning to be prepared for later testing. On one hand, cognitive offloading might not generally have detrimental effects on memory for the offloaded information but might even have beneficial long-term consequences when participants are explicitly instructed to memorise the studied material. For instance, it is commonly argued that cognitive offloading releases internal cognitive resources (Kirsh, 2010). When offloading information that otherwise would need to be stored in working memory, the released internal cognitive resources can be referred to as individuals’ unused working memory capacity. Such released cognitive resources might in turn serve to gain a deeper processing of the remaining task-relevant information and, therefore, improve memory (Craik & Lockhart, 1972) as well as learning (Salomon, 1990; Sweller et al., 1998). Consequently, someone who aims at acquiring long-term memory might strategically use cognitive offloading to form stronger memory representations.

On the other hand, however, if the released cognitive resources (due to offloading) cannot be directed to the remaining task-relevant information even with the explicit instruction to memorise the stimuli, the availability of technical tools might provide a risk for subsequent memory performance, as offloading decreases the overall amount of internal information processing and elaboration. In this case, strategic considerations should minimise cognitive offloading to create desirable difficulties (i.e., conditions of learning that make it more difficult but increase learning; Bjork & Bjork, 2011). Introducing desirable difficulties by using more memory-intense strategies and less offloading might be more demanding but might also enhance learning and memory (Bjork & Bjork, 2011). Therefore, in our research we also directly tested whether the awareness of a follow-up memory test alters offloading behaviour as well as its consequences.

## Present research

In the present research, we systematically investigated how cognitive offloading affects subsequent memory for the offloaded information. In Experiment 1, we aimed at demonstrating the proposed trade-off between immediate beneficial effects of offloading on task processing and subsequent detrimental effects of cognitive offloading on memory. This experiment is similar to the experiments reported by Morgan et al. (2009; see also Morgan et al., 2013; Waldron et al., 2007) with the difference that our memory test was delayed substantially following the completion of the Pattern Copy Task, whereas Morgan et al. (2009) presented the memory test in between the trials of the Pattern Copy Task. Furthermore, our memorised information consisted of more naturalistic stimuli (i.e., images of real-world objects) rather than coloured squares. Nevertheless, due to the high similarity across the studies, our first experiment could be considered to be a conceptual replication of the findings of Morgan et al. (2009).

In Experiments 2 and 3, we studied how awareness of the upcoming memory test, and thus the goal to foster learning, influences offloading behaviour as well as its consequences for memory. The number of stimuli in the Pattern Copy Task exceeds working memory capacity. Therefore, it cannot be solved without at least some degree of cognitive offloading. This allowed us to test whether the detrimental effects of offloading also arise under conditions in which participants know that they will have to recall the offloaded information at a later point in time. More specifically, we tested two competitive hypotheses. On one hand, released cognitive resources due to offloading might be used to build long-term memory representations. If this is the case, offloading behaviour would not be detrimental to long-term memory acquisition. On the other hand, if devoting released resources to the formation of long-term memory is not possible, offloading behaviour would be detrimental for long-term memory acquisition. In this case, offloading behaviour should be minimised to create desirable difficulties that improve learning. To distinguish between these hypotheses, we manipulated the awareness of a follow-up memory test and investigated whether test awareness alters the use and the effects of cognitive offloading. In Experiment 2, the participants performed the Pattern Copy Task under free-choice conditions (i.e., the participants could freely choose whether to offload or not). In the final Experiment 3, we compared this free-choice conditions with a condition in which we enforced offloading to the maximum extent.

## Experiment 1

This experiment focused on demonstrating the proposed trade-off between immediate task performance and the formation of memory representations. Our participants completed a version of the Pattern Copy Task which clearly exceeds working memory capacity. Therefore, all participants had to rely on offloading behaviour although the amount of offloading might have varied between them. In this task, increasing offloading behaviour reduced the amount of information that needed to be handled simultaneously within working memory. Importantly, however, each participant had to process every unit of information to solve the task. Beyond measuring individual offloading behaviour, we manipulated the temporal costs of offloading to alter the amount of cognitive offloading (i.e., higher costs of externalisation induce a stronger reliance on internal resources). Following a retention interval at the end of the experiment, the participants completed an unexpected memory test. We predicted that more offloading increases immediate task performance with regard to efficiency (speed and accuracy), but impairs the formation of memory representations.

Except for the retention interval exceeding the duration of working memory as well as the more naturalistic objects to memorise, our task and procedure are similar to the experiments reported in Morgan et al. (2009). Due to these similarities, our first experiment could be considered to reflect a conceptual replication of the previously established result pattern. Nevertheless, we consider it important to replicate previous findings with novel variants of tasks to prove the generalisability of the concepts under study as well as the suitability of the present materials and procedure.

### Method

This experiment was preregistered at the Open Science Framework (OSF; https://osf.io/n64cd). In addition, all materials, data, and analysis scripts are available at https://osf.io/vmgd4/. Analyses that are labelled as “exploratory” within the results sections were not formally preregistered.

#### Participants

Our final sample consisted of 172 students (131 females; 18–47 years) recruited at the University of Tübingen. The participants received course credit or a financial compensation of €8 for 1 hr of their time. The study was approved by the ethical board of the Leibniz-Institut für Wissensmedien, Tübingen, Germany (Nr.: LEK 2017/059), and all participants provided written informed consent prior to testing. The sample size was preregistered and intended to achieve a statistical power of (1 − β) = .90 at medium effect sizes of *f* = 0.25.^1^ The participants were randomly assigned to one of the two conditions (*n* = 86 per condition). According to the preregistered exclusion criteria, data from participants with missing data (16), failures in complying with the instructions (5), a priori awareness of the surprise memory test (14),^2^ too many errors in the working memory tests to compute capacity (1), or too large deviations (±3 *SD*) in any dependent variable of the Pattern Copy Task or the memory test (15) were replaced. Each participant performed the experiment individually in a testing room.

#### Apparatus

All tasks were controlled by PsychoPy scripts (Peirce, 2007) running on 12.3″ Microsoft Surface Pro tablets (2,736 × 1,824 pixels; touch served as input device) lying flat on the table at a viewing distance of approximately 36 cm.

#### Tasks and stimuli

##### Pattern Copy Task

**Figure 1.**
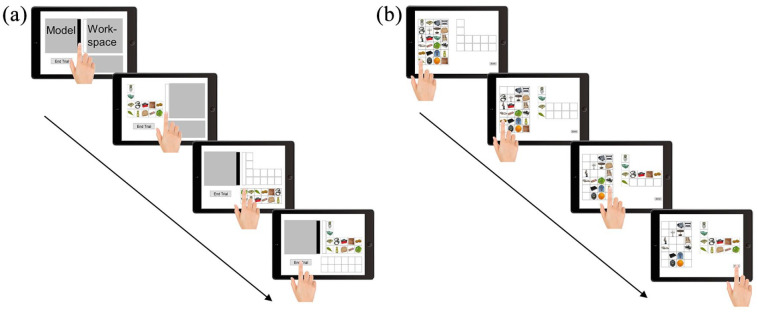
Illustration of the Pattern Copy Task and memory test. (a) Pattern Copy Task. Participants copied the layout of images from the model window (left) to a workspace window (right). Importantly, only one of the windows was visible at a time. This task measures offloading behaviour (i.e., the amount of simultaneously copied images) as well as the immediate task performance (i.e., speed and accuracy). On a group level, we induced offloading behaviour by manipulating the temporal costs of accessing the model window. (b) Memory test. The participants restored the configurations of the Pattern Copy Task from memory. (Tablet frame designed by Freepik; Index finger designed by Jannoon028/Freepik.)

This task was designed to measure offloading behaviour (Ballard et al., 1995). The participants dragged-and-dropped 12 images of distinct objects (each 3.5° × 3.5°; selected from the *Bank of standardized stimuli*; Brodeur et al., 2010, 2014) from a resource window in the lower right screen to a workspace window in the upper right screen. The aim was to replicate a layout of the same objects from an identically shaped model window in the upper left area of the screen (see Figure 1a). At any time, either the model or the workspace window was visible while the other window was covered. The participants were able to open the model window by using a slider on the right side of that window and the workspace window by touching a bar left to it. For one-half of the participants, opening the model window resulted in a delay of 2 s (for which the slider turned red). Therefore, these participants had to wait to open the model window (lockout condition), whereas the remaining half of the participants could open the model window immediately (no lockout condition; see for example, Gray et al., 2006; Grinschgl, Meyerhoff, & Papenmeier, 2020; for a similar manipulation). The participants were allowed to switch between the two windows as often as they wanted. At the beginning of each trial, grey masks covered all windows, and the participants could decide which window to open first. After rebuilding the 12 images, they pressed a button in the lower left area of the screen to continue. There were 20 trials with distinct spatial arrangements of the patterns to be copied, preceded by one practice trial. The images showed (coloured) common objects from everyday life such as kitchenware, clothing, or food products (for a further description see Brodeur et al., 2010, 2014; all images we used are available on https://osf.io/vmgd4/). A new set of images was selected randomly without replacement from a collection of 480 objects (240 for the Pattern Copy Task; 240 additional distractors for the memory test) for each trial. The sets of images were counterbalanced across conditions (i.e., one participant from each condition saw the same sets of objects in the same order).

As proxies for cognitive offloading, we analysed the number of openings of the model window (i.e., openings of the model window), the duration of the very first opening (i.e., initial encoding duration), and the number of correctly copied items following the first opening of the model window (i.e., initially correctly copied items; only the first opening within a trial is independent of preceding openings). More pronounced offloading behaviour is indicated by opening the window more often as well as shorter initial encoding durations and fewer initially correctly copied objects. Furthermore, as immediate task performance, we measured the trial duration (corrected for the 2-s lockout times) as well as the number of errors. The number of errors refers to the number of incorrectly rebuild images at the end of a trial. Higher trial duration and more errors indicate lower immediate task performance.

##### Memory test

This task was designed to assess memory performance for the objects handled in the Pattern Copy Task. The participants’ task was to restore each of the 20 unique spatial arrangements of objects from the Pattern Copy Task from their memory (see Figure 1b). In each trial, one of the grids was presented in the centre of the screen. Next to the grid, the original 12 images of this particular grid were presented intermixed with 12 new distractor images from the same database that had not been presented before (each 3.5° × 3.5°). The participants were instructed to rebuild the original arrangement by drag-and-dropping the correct images to the correct locations within the arrangement. As proxies for memory performance, we calculated the proportion of correctly restored identity-location bindings (i.e., correct image at the correct location). We calculated this proportion relative to both the reproduced pattern in the Pattern Copy Task (i.e., identity-location bindings corrected) as well as the original pattern within the Pattern Copy Task (i.e., identity-location bindings). We conducted this two-fold analysis to exclude the possibility of our results emerging from carry-over effects of initial copy errors. Furthermore, we calculated the proportion of correctly restored identities (i.e., correct image in any location; identity).

##### Additional tests

We implemented a retention interval between the Pattern Copy Task and the memory test. During this interval, our participants completed two additional working memory tests as a proxy for their working memory capacity without offloading. We chose to implement these tasks as the impact of working memory capacity on offloading has been controversially discussed in previous research (e.g., Morrison & Richmond, 2020; Risko & Dunn, 2015). We therefore wanted to make sure that observed effects in offloading behaviour and memory performance cannot simply be explained in terms of a priori differences between the experimental groups. In the first test, the participants had to reconstruct visual patterns of coloured squares (*Visual Patterns Test*; adapted from Della Sala et al., 1999). In the second task, the participants had to reconstruct a temporal sequence of spatial locations (*Corsi Blocks Task*; adapted from Milner, 1971). Both tests followed an adaptive staircase of difficulty (starting with 2 objects, +1 *object if correct*, −1 *object if false*, minimum of two objects), and we analysed the set size of the last 10 correctly solved trials (out of a total of 30 trials).

### Results

In line with our preregistered hypothesis, the participants in the no lockout condition performed more cognitive offloading and had a higher efficiency in immediate task performance, but subsequently showed less accurate memory performance. Furthermore, individual differences in offloading behaviour revealed the same pattern of results.

#### Cognitive offloading

For all three proxies of cognitive offloading, *t*-tests for independent samples indicated more offloading in the no lockout than in the lockout condition (see Table 1). The participants in the no lockout condition opened the model window more frequently, *t*(170) = 7.55, *p* < .001, $\eta_{p}^{2}=.25$, 95% CI = [0.15, 0.35], showed a shorter initial encoding of the model window, *t*(170) = −3.87, *p* < .001, $\eta_{p}^{2}=.08$, 95% CI = [0.02, 0.17], and copied fewer items correctly within the first copy cycle, *t*(170) = −4.13, *p* < .001, $\eta_{p}^{2}=.09$, 95% CI = [0.03, 0.18], in comparison to those participants in the lockout condition.

**Table 1.** Means and standard errors of dependent variables in Experiment 1.

	No lockout		Lockout	
	*M*	*SE*	*M*	*SE*
Cognitive offloading				
Openings of the model window	3.61	0.15	2.31	0.09
Initial encoding duration (s)	13.99	0.89	20.14	1.31
Initially correctly copied items	5.45	0.28	7.22	0.32
Immediate task performance				
Trial duration (s)	49.33	1.19	55.06	1.25
Errors	0.19	0.04	0.21	0.03
Memory performance				
Identity	10.55	0.11	10.93	0.09
Identity-location bindings	5.91	0.30	7.07	0.33
Identity-location bindings (corrected)	5.91	0.30	7.08	0.33
Working memory capacity				
Visual Patterns Test	3.84	0.05	3.89	0.05
Corsi Blocks Task	4.76	0.07	4.73	0.08

SE: standard error.

Cognitive offloading (openings of the model window and initial encoding duration), immediate task performance, and working memory capacity refer to open-ended count or time data. Initially correctly copied items and memory performance refer to count data with a maximum of 12.

#### Immediate task performance

Two *t*-tests for independent samples showed that participants in the no lockout condition solved the Pattern Copy Task more efficiently than participants in the lockout condition (see Table 1; values are corrected for lockout times). In other words, they solved the task in less time, *t*(170) = −3.31, *p* = .001, $\eta_{p}^{2}=.06$, 95% CI = [0.01, 0.14], without decrements in accuracy, *t*(170) = −0.37, *p* = .709, $\eta_{p}^{2}<.01$, 95% CI = [0.00, 0.03]. With on average below one error per trial in both groups, performance in terms of accuracy was at ceiling.

#### Memory performance

While being more efficient in immediate task performance, the participants in the no lockout condition performed less accurately in the unexpected memory test (see Table 1). A series of *t*-tests for independent samples confirmed that the participants in the no lockout condition remembered the identity of the images, *t*(170) = –2.76, *p* = .006, $\eta_{p}^{2}=.04$, 95% CI = [0.003, 0.12], as well as uncorrected, *t*(170) = –2.59, *p* = .010, $\eta_{p}^{2}=.04$, 95% CI = [0.002, 0.11], and corrected (i.e., for errors in the Pattern Copy Task) identity-location bindings, *t*(170) = –2.63, *p* = .009, $\eta_{p}^{2}=.04$, 95% CI = [0.002, 0.11], worse than the participants in the lockout condition. In other words, a high degree of cognitive offloading in the no lockout condition negatively affected subsequent memory accuracy. This finding was further emphasised by an exploratory analysis of individual differences,^3^ which showed strong correlations between offloading behaviour and memory accuracy (see Table 2 and Figures S1.1, S1.2, and S1.3 in the electronic supplementary material [ESM] for the corresponding scatter plots). In both experimental conditions, Pearson’s correlations showed that increasing offloading behaviour was clearly associated with decreasing memory accuracy across all proxies for offloading and memory, all |*r|*s(84) ⩾ .51, all *p*s < .001.

**Table 2.** Pearson’s correlations between cognitive offloading and memory performance in Experiment 1.

	Identity	
	No lockout	Lockout
Openings of the model window	−.54***	−.61***
Initial encoding duration (s)	.51***	.63***
Initially correctly copied items	.68***	.70***
	Identity-location bindings	
	No lockout	Lockout
Openings of the model window	−.58***	−.69***
Initial encoding duration (s)	.65***	.67***
Initially correctly copied items	.85***	.79***
	Identity-location bindings (corrected)	
	No lockout	Lockout
Openings of the model window	−.58***	−.69***
Initial encoding duration (s)	.65***	.67***
Initially correctly copied items	.85***	.79***

* *p* < .05, **p < .01, ***p < .001.

#### Mediation analyses

An alternative explanation for improved memory performance in the lockout condition is that this benefit does not stem from the reduction in offloading behaviour but that participants take advantage of the 2-s lockout for additional rehearsal in this condition. To provide evidence that reduced offloading behaviour indeed increased memory performance, we conducted a set of exploratory mediation analyses. Within these analyses, we probed whether our independent variable, temporal lockout, directly affected subsequent memory performance, or whether this relationship was mediated by cognitive offloading (see Table 3). We observed a mediated effect of the predictor lockout (no lockout/lockout) via cognitive offloading (mediator) on all three memory variables (identity, identity-location bindings, identity-location bindings corrected), all mediated effects ⩾0.29, all *p*s < .001. In fact, the mediations substantially reduced all direct effects of the predictor on the memory performance measures, all |direct effects| ⩽0.86, all *p*s > .040. Only in two analyses (out of nine), did the direct effect remain significant. This indicates that the reduction in offloading behaviour accompanying temporal lockouts rather than the lockout manipulation itself enhanced memory performance.

**Table 3.** Mediation analyses with the predictor lockout (no lockout/lockout), mediator cognitive offloading, and outcome memory performance in Experiment 1.

	Identity							
	Mediated effect	95% CI	Direct effect	95% CI	Total effect	95% CI	Prop. mediated	95% CI
Openings of the model window	0.58***	[0.40, 0.80]	−0.20	[−0.42, 0.06]	0.38**	[0.09, 0.63]	1.52**	[1.16, 17.45]
Initial encoding duration (s)	0.29***	[0.16, 0.46]	0.09	[−0.15, 0.31]	0.38**	[0.09, 0.63]	0.77**	[0.55, 4.14]
Initially correctly copied items	0.39***	[0.21, 0.60]	−0.01	[−0.21, 019]	0.38**	[0.09, 0.63]	1.02**	[0.76, 5.45]
	Identity-location bindings							
Openings of the model window	2.01***	[1.53, 2.58]	−0.86*	[−1.61, −0.01]	1.16*	[0.29, 2.00]	1.74*	[1.21, 10.86]
Initial encoding duration (s)	1.13***	[0.59, 1.71]	0.02	[−0.63, 0.68]	1.16*	[0.29, 2.00]	0.98*	[0.72, 4.70]
Initially correctly copied items	1.50***	[0.83, 2.26]	−0.34	[−0.87, 0.21]	1.16*	[0.29, 2.00]	1.29*	[1.01, 7.46]
	Identity-location bindings (corrected)							
Openings of the model window	2.01***	[1.53, 2.59]	−0.84*	[−1.59, −0.01]	1.17*	[0.28, 2.01]	1.71*	[1.21, 19.16]
Initial encoding duration (s)	1.13***	[0.59, 1.71]	0.04	[−0.61, 0.71]	1.17*	[0.28, 2.01]	0.96*	[0.72, 7.65]
Initially correctly copied items	1.50***	[0.83, 2.25]	−0.33	[−0.86, 0.23]	1.17*	[0.28, 2.01]	1.28*	[1.00, 9.75]

CI: confidence interval.

All mediation analyses were conducted with a bootstrapping procedure (1,000 simulations) using the package “mediation” in R (Tingley et al., 2014).

* *p* < .05, **p < .01, ***p < .001.

#### Working memory capacity

Two *t*-tests for independent samples confirmed that there were no group differences between participants in the lockout and the no lockout condition with regard to memory capacity as measured by the Visual Patterns Test, *t*(170) = −0.56, *p* = .576, $\eta_{p}^{2}<.01$, 95% CI = [0.00, 0.03], BF_01_ = 5.24^4^ and the Corsi Blocks Task, *t*(170) = 0.23, *p* = .820, $\eta_{p}^{2}<.01$, 95% CI = [0.00, 0.02], BF_01_ = 5.92 (see Table 1). Exploratory correlational analysis of working memory capacity, cognitive offloading, and subsequent memory performance are available in Table S1 of the ESM.

## Experiment 2

The observation that more cognitive offloading impairs the formation of memory representations in Experiment 1 arises from an incidental experimental set-up (i.e., participants were not aware of the memory test while performing the Pattern Copy Task). Whereas this finding shows impaired memory for situations with implicit formation of new memory representations, it does not necessarily transfer to scenarios in which participants explicitly aim at forming memory representations for subsequent testing. In the second experiment, we investigated how awareness of the subsequent memory test alters offloading behaviour as well as the formation of memory representations. To study this, one-half of the participants remained uninformed with regard to the subsequent memory test whereas we explicitly informed the other half of the participants that they would have to complete a memory test after the Pattern Copy Task. In addition, we manipulated the temporal costs of offloading. With this set-up, we tested two competing hypotheses. The first hypothesis is that cognitive offloading releases internal cognitive resources and that test awareness is necessary to devote these released resources to the formation of memory representations. The second hypothesis is that released resources do not contribute to the formation of memory representations. In this case, the participants might rely more on their own internal encoding strategies and thus avoid cognitive offloading to foster long-term learning (i.e., a desirable difficulty).

### Method

This experiment was preregistered at the OSF (https://osf.io/pb89m). All materials, data, and analysis scripts are available at https://osf.io/ke9dj/. All methods of this experiment were identical to Experiment 1 with the exceptions described in this section.

#### Participants

The final sample size consisted of 172 new students (137 females; 18–35 years), who were randomly assigned to the four conditions (*n* = 43 per condition). According to the preregistered exclusion criteria, we replaced data from participants with missing data (1), a priori awareness of the surprise memory test (19),^5^ or too large deviations (±3 *SD*) in any dependent variable of the Pattern Copy Task or the memory test (4). The preregistered exclusion criteria were identical to those of Experiments 1 and 3.

#### Tasks and stimuli

##### Pattern Copy Task

For one-half of the participants, the instructions included information announcing the upcoming memory test at the end of the experiment (translated from German: “Please note: Following the task, you will have to take a memory test which will test your memory of the presented patterns of images.”). In addition, before the first trial of the Pattern Copy Task, the participants were reminded about the memory test (translated from German: “Please try to remember the pictures and patterns as well as possible, as a recognition test will be carried out after this test.”). For the other half of the participants, none of this information about the upcoming memory test was included in the instructions. The original instructions for each condition as well as the tests themselves are available at https://osf.io/ke9dj/.

As the focus of this experiment was on the effect of offloading on subsequent memory rather than immediate task performance, we aimed to ensure that all participants had the same chance to remember the initial pattern of objects. Therefore, the participants had to solve the Pattern Copy Task correctly in this experiment. Consequently, the participants could not proceed to the next trial without correctly replicating the layout from the model window. Nevertheless, we will still report trial duration as an index of immediate task performance. The patterns of this experiment consisted of eight images only (these images are a subset of the images used in Experiment 1; see https://osf.io/ke9dj/).^6^

##### Memory test

The layout within the memory test was slightly different. The eight original and eight distractor images were presented below instead of next to the corresponding grid. As we used fewer images (compared to the other experiments), this adapted layout appeared more user-friendly.

### Results

Matching the results of Experiment 1, more offloading resulted in faster task processing but less accurate memory performance. Interestingly, participants who knew about the subsequent memory test reduced offloading behaviour and subsequently showed an improved memory performance.

#### Cognitive offloading

We analysed all three proxies for cognitive offloading using 2 × 2 between-subjects analysis of variances (ANOVAs) with lockout and announcement of the memory test as the independent variables. Each proxy of cognitive offloading indicated more offloading in the no lockout than in the lockout condition (see Table 4). The participants in the no lockout condition opened the model window more frequently, *F*(1, 168) = 37.25, *p* < .001, $\eta_{p}^{2}=.18$, 95% CI = [0.09, 0.28], showed a shorter initial encoding of the model window, *F*(1, 168) = 15.58, *p* < .001, $\eta_{p}^{2}=.09$, 95% CI = [0.02, 0.17], and copied fewer items correctly within the first copy cycle, *F*(1, 168) = 24.50, *p* < .001, $\eta_{p}^{2}=.13$, 95% CI = [0.05, 0.22], than the participants in the lockout condition. Furthermore, across all variables, the participants in the uninformed condition offloaded more than the participants in the informed condition. Thus, the participants in the uninformed condition opened the model window more frequently, *F*(1, 168) = 10.31, *p* = .002, $\eta_{p}^{2}=.06$, 95% CI = [0.009, 0.14], showed a shorter initial encoding of the model window, *F*(1, 168) = 19.29, *p* < .001, $\eta_{p}^{2}=.10$, 95% CI = [0.03, 0.19], and copied fewer items correctly within the first copy cycle, *F*(1, 168) = 12.21, *p* < .001, $\eta_{p}^{2}=.07$, 95% CI = [0.01, 0.15], than the participants in the informed condition. No interactions between the conditions were found, all *Fs*(1, 168) ⩽ 1.83, all *ps* ⩾ .178.

**Table 4.** Means and standard errors of dependent variables in Experiment 2.

	No lockout				Lockout			
	Uninformed		Informed		Uninformed		Informed	
	*M*	*SE*	*M*	*SE*	*M*	*SE*	*M*	*SE*
Cognitive offloading								
Openings of the model window	2.49	0.15	1.98	0.13	1.65	0.09	1.45	0.07
Initial encoding duration (s)	9.52	0.80	14.08	1.18	13.63	1.01	17.83	0.95
Initially correctly copied items	4.73	0.27	5.72	0.28	6.09	0.25	6.87	0.19
Immediate task performance								
Trial duration (s)	28.85	0.99	32.73	1.05	33.76	1.24	36.79	1.15
Memory performance								
Identity	6.99	0.12	7.32	0.09	7.27	0.14	7.66	0.04
Identity-location bindings	4.20	0.29	5.17	0.29	5.24	0.31	6.28	0.19
Working memory capacity								
Visual Patterns Test	3.88	0.08	3.95	0.07	3.76	0.09	3.95	0.09
Corsi Blocks Task	4.61	0.10	4.79	0.09	4.60	0.09	4.87	0.10

SE: standard error.

In this experiment, initially correctly copied items and memory performance refer to count data with a maximum of 8.

#### Immediate task performance

With regard to immediate task performance, more cognitive offloading came along with faster task processing (see Table 4). We confirmed this with an exploratory 2 × 2 between-subjects ANOVA with lockout and announcement of the memory test as the independent variables and trial duration as the dependent variable. The participants in the no lockout condition completed the trials faster than the participants in the lockout condition, *F*(1, 168) = 16.24, *p* < .001, $\eta_{p}^{2}=.08$, 95% CI = [0.02, 0.18]. Furthermore, the participants in the uninformed condition completed the trials faster than the participants in the informed condition, *F*(1, 168) = 9.62, *p* = .005, $\eta_{p}^{2}=.05$, 95% CI = [0.01, 0.13]. The interaction between both conditions was not significant, *F*(1, 168) = 0.14, *p* = .706.

#### Memory performance

While offloading more within the Pattern Copy Task, the participants in the no lockout condition as well as the uninformed condition performed less accurately in the memory test (see Table 4). We analysed both proxies for memory accuracy using 2 × 2 between-subjects ANOVAs with lockout and announcement of the memory as the independent variables. The participants in the no lockout condition performed less accurately in identifying the images, *F*(1, 168) = 8.29, *p* = .004, $\eta_{p}^{2}=.05$, 95% CI = [0.005, 0.12]), as well as in retrieving identity-location bindings, *F*(1, 168) = 15.09, *p* < .001, $\eta_{p}^{2}=.08$, 95% CI = [0.02, 0.17], in comparison to the participants in the lockout condition. Furthermore, uninformed participants performed less accurately in identifying the images, *F*(1, 168) = 11.58, *p* < .001, $\eta_{p}^{2}=.06$, 95% CI = [0.01, 0.14], and the identity-location bindings, *F*(1, 168) = 13.20, *p* < .001, $\eta_{p}^{2}=.07$, 95% CI = [0.02, 0.16], than informed participants. There was no interaction between the investigated conditions, all *Fs*(1, 168) ⩽ 0.08, all *ps* ⩾ .773. These findings were further supported by the exploratory analyses of individual differences which showed correlations between offloading behaviour and memory accuracy (see Table 5 and ESM Figures S2.1 and S2.2). Increasing offloading behaviour was associated with decreasing memory accuracy.

**Table 5.** Pearson’s correlations between cognitive offloading and memory performance in Experiment 2.

	Identity			
	No lockout		Lockout	
	Uninformed	Informed	Uninformed	Informed
Openings of the model window	−.70***	−.47**	−.55***	−.37*
Initial encoding duration (s)	.53***	.47**	.40**	.29
Initially correctly copied items	.67***	.57***	.55***	.42**
	Identity-location bindings			
	No lockout		Lockout	
	Uninformed	Informed	Uninformed	Informed
Openings of the model window	−.53***	−.58***	−.61***	−.64***
Initial encoding duration (s)	.45**	.60***	.35*	.47**
Initially correctly copied items	.53***	.69***	.65***	.68***

* *p* < .05, **p < .01, ***p < .001.

#### Mediation analyses

As in Experiment 1, we conducted a set of exploratory mediation analyses to provide evidence that the reduction of offloading behaviour increased memory accuracy in the lockout conditions (see Table 6). We observed a mediated effect of the predictor lockout (no lockout/lockout) via cognitive offloading (mediator) on all memory variables (identity, identity-location bindings), all mediated effects ⩾0.18, all *p*s ⩽ .001, all |direct effects|⩽ 0.53, all *p*s ⩾ .046. In only one case (out of six), the direct effect remained significant while the mediating factor still appears to be the stronger predictor. Hence, the lockout manipulation influenced subsequent memory performance by affecting offloading behaviour. Furthermore, regarding the announcement of the upcoming memory test (informed/not informed), we also observed a mediation of the effect of announcement via cognitive offloading, all |mediated effects|⩾ 0.18, all *p*s ⩽ .004, rather than a direct effect on subsequent memory performance, all |direct effects|⩽ 0.50, all *p*s ⩾ .026 (see Table 7). In two analyses (out of six), the direct effect remained significant; however, the mediated effect appears to be stronger overall.

**Table 6.** Mediation analyses with the predictor lockout (no lockout/lockout), mediator cognitive offloading, and outcome memory performance in Experiment 2.

	Identity							
	Mediated effect	95% CI	Direct effect	95% CI	Total effect	95% CI	Prop. mediated	95% CI
Openings of the model window	0.36***	[0.22, 0.52]	−0.06	[−0.28, 0.14]	0.30**	[0.09, 0.51]	1.20**	[0.78, 11.38]
Initial encoding duration (s)	0.18***	[0.09, 0.33]	0.12	[−0.09, 0.32]	0.30**	[0.09, 0.51]	0.61**	[0.36, 6.10]
Initially correctly copied items	0.30***	[0.17, 0.47]	−0.003	[−0.22, 0.19]	0.30**	[0.09, 0.51]	1.01**	[0.67, 7.84]
	Identity-location bindings							
Openings of the model window	0.99***	[0.68, 1.32]	0.09	[−0.42, 0.68]	1.08***	[0.49, 1.61]	0.92***	[0.64, 2.16]
Initial encoding duration (s)	0.55***	[0.26, 0.90]	0.53*	[0.03, 1.03]	1.08***	[0.49, 1.61]	0.51***	[0.30, 1.06]
Initially correctly copied items	0.90***	[0.50, 1.31]	0.17	[−0.31, 0.64]	1.08***	[0.49, 1.61]	0.84***	[0.58, 1.73]

CI: confidence interval.

* *p* < .05, **p < .01, ***p < .001.

**Table 7.** Mediation analyses with the predictor announcement of the memory test (informed/uninformed), mediator cognitive offloading, and outcome memory performance in Experiment 2.

	Identity							
	Mediated effect	95% CI	Direct effect	95% CI	Total effect	95% CI	Prop. mediated	95% CI
Openings of the model window	−0.18**	[−0.30, −0.06]	−0.18*	[−0.35, −0.02]	−0.36***	[−0.55, −0.15]	0.49**	[0.24, 0.94]
Initial encoding duration (s)	−0.20***	[−0.32, −0.11]	−0.16	[−0.34, 0.03]	−0.36***	[−0.55, −0.15]	0.56***	[0.37, 1.47]
Initially correctly copied items	−0.21***	[−0.34, −0.08]	−0.15	[−0.32, 0.01]	−0.36***	[−0.55, −0.15]	0.58***	[0.33, 1.13]
	Identity-location bindings							
Openings of the model window	−0.50**	[−0.82, −0.17]	−0.50*	[–0.98, −0.07]	−1.01***	[−1.57, −0.46]	0.50**	[0.24, 0.97]
Initial encoding duration (s)	−0.62***	[−1.02, −0.33]	−0.39	[−0.92, 0.18]	−1.01***	[−1.57, −0.46]	0.61***	[0.34, 1.53]
Initially correctly copied items	−0.63***	[−1.03, −0.26]	−0.38	[−0.85, 0.05]	−1.01***	[−1.57, −0.46]	0.62***	[0.35, 1.19]

CI: confidence interval.

* *p* < .05, **p < .01, ***p < .001.

#### Working memory capacity

We analysed both proxies for working memory capacity using 2 × 2 between-subjects ANOVAs with lockout and announcement of the memory test as the independent variables. We did not find any main effects or interactions in working memory capacity for identity-location bindings as measured by the Visual Patterns Test, all *F*s(1, 168) ⩽ 2.38, all *p*s ⩾ .13, all BF_01_s ⩾ 2.00^7^ (see Table 4). In the Corsi Blocks Task, the participants in the uninformed condition showed a lower working memory capacity for temporal sequence of spatial locations, than the participants in the informed condition, *F*(1, 168) = 4.85, *p* = .029, $\eta_{p}^{2}=.03$, 95% CI = [0.00, 0.09], BF_01_ = 0.64. However, performance in the Corsi Blocks Task was uncorrelated with cognitive offloading, all |*r|s*(41) ⩽ .21, all *p*s > .173, as well as memory performance in the main task, all |*r|s*(41) ⩽ .21, all *p*s > .166 (exploratory analyses; see Table S2 in the ESM). Therefore, we will not address this discrepancy any further. Nevertheless, please note, the Corsi Blocks Task was performed after the Pattern Copy Task to serve as a retention internal. Thus, it cannot be considered independent of our experimental design and differences in Corsi Blocks capacity might have been induced by our experimental manipulations. There were no other group differences or interactions in the Corsi Blocks Task, all *F*s(1, 168) ⩽ 0.12, all *p*s ⩾ .66, all BF_01_s ⩾ 2.79 (see Table 4).

## Experiment 3

Experiments 1 and 2 both showed that cognitive offloading was associated with reduced subsequent memory performance. When participants were aware of the upcoming memory test, however, they seem to reduce offloading behaviour to foster long-term memory (Experiment 2). This finding suggests that cognitive resources which are released by offloading are rather “lost” than devoted to the acquisition of memory representations. In this third experiment, we further pursue on this finding by probing its’ generality and/or boundaries. Therefore, we manipulated whether participants were allowed to freely choose their offloading behaviour as in the previous experiments (choice condition) or whether they were forced to offload to a maximum extent (forced condition). Identically to Experiment 2, we further manipulated whether the participants were aware of the upcoming memory test (informed condition) or not (uninformed condition). If cognitive offloading is harmful for the formation of memory representations in general, being aware of the upcoming memory test should have no beneficial effect when the participants are forced to offload maximally. Furthermore, the condition with the free-choice offloading also allows us to reinvestigate the interesting finding of Experiment 2, namely, that participants who were aware of the subsequent memory test rather avoided offloading to improve memory performance.

### Method

We preregistered this experiment at OSF (https://osf.io/4ye2c). Furthermore, all materials, data, and analysis scripts are available at https://osf.io/k6t7q/. All methods of this experiment were identical to Experiment 1 and 2 with the exceptions described in this section.

#### Participants

Our final sample consisted of 172 new students (136 females; 18–66 years). Based on our preregistered exclusion criteria, we replaced data of participants with missing data (12), a priori awareness of the surprise memory test (27),^8^ or too large deviations (±3 *SD*) in any preregistered dependent variable of the Pattern Copy Task or the memory test (6). The preregistered exclusion criteria were identical to Experiments 1 and 2. This experiment was conducted in a group setting with a maximum of four participants at once. The testing room was divided into separate chambers by movable walls so that the participants could not see each other during the study.

#### Tasks and stimuli

##### Pattern Copy Task

Orthogonally to the manipulation of memory test awareness (see also Experiment 2), we manipulated whether participants could freely choose their offloading behaviour (choice condition) or whether they were forced to offload to a maximum extent (forced condition). The choice condition was identical to the no lockout condition in Experiments 1 and 2. In the novel forced condition, the participants were only allowed to rebuild a single object in the workspace window within each copy cycle of the Pattern Copy Task. Therefore, the participants had to change between the model and the workspace window at least 12 times to rebuild the 12 images in this condition. In this experiment, the participants could open the model window by just clicking on the bar next to it (instead of using a slider as in Experiments 1 and 2). For all conditions, the Pattern Copy Task had to be solved correctly (see also Experiment 2). As proxies of cognitive offloading, we preregistered the number of openings of the model window and the number of correctly copied items after the first opening.^9^

##### Memory test

The memory test was identical to Experiment 1.

### Results

In line with our previous findings, we observed that cognitive offloading was detrimental to subsequent memory performance when participants were unaware of the upcoming memory test. Nonetheless, when participants were aware of the memory test, the detrimental effects of cognitive offloading on memory performance were less pronounced. In particular, the participants who were forced to offload to a maximum extent recovered remarkably from the lack of memory representations relative to their uninformed counterparts.

#### Cognitive offloading

Because one-half of our participants were forced to offload to a maximum extent, we analysed offloading behaviour only for the participants performing the task under the free-choice conditions. First, we confirmed that the participants in the choice condition actually offloaded less extensively than maximum offloading. Therefore, we conducted one-sample *t*-tests for the informed and uninformed conditions. In both choice (sub-)conditions, the participants offloaded less than they maximally could (see Table 8). We observed fewer openings of the model window in the choice/uninformed condition, *t*(42) = −39.33, *p* *<* .001, *d* = 6.07, 95% CI = [4.75, 7.38], as well as the choice/informed condition, *t*(42) = −43.18, *p* *<* .001, *d* = 6.66, 95% CI = [5.22, 8.10], compared to μ = 12 (i.e., the minimum amount of opening the model window in the forced condition). In addition, we also observed that the participants copied more items initially correctly in the choice/uninformed condition, *t*(42) = 11.01, *p* *<* .001, *d* = 1.69, 95% CI = [1.23, 2.17], and the choice/informed condition, *t*(42) = 12.89, *p* *<* .001, *d* = 1.98, 95% CI = [1.47, 2.50], compared to μ = 1 (i.e., the maximum amount of copied items in forced condition per opening). Therefore, consistent with the previous experiments, the participants in the choice condition relied on offloading, but they did not offload maximally.

**Table 8.** Means and standard errors of dependent variables in Experiment 3.

	Choice				Forced			
	Uninformed		Informed		Uninformed		Informed	
	*M*	*SE*	*M*	*SE*	*M*	*SE*	*M*	*SE*
Cognitive offloading								
Openings of the model window	3.68	0.21	3.79	0.19	12.36	0.04	12.29	0.03
Initially correctly copied items	4.96	0.36	4.61	0.28	0.97	0.01	0.98	0.01
Immediate task performance								
Trial duration (s)	42.56	1.48	45.38	2.98	35.35	1.59	45.36	2.37
Memory performance								
Identity	9.97	0.18	10.20	0.13	8.17	0.17	9.34	0.22
Identity-location bindings	4.69	0.41	5.18	0.43	1.84	0.18	4.18	0.45
Working memory capacity								
Visual Patterns Test	3.87	0.06	3.97	0.07	3.81	0.08	3.87	0.08
Corsi Blocks Task	4.59	0.09	4.71	0.09	4.65	0.12	4.94	0.09

SE: standard error.

In this experiment, initially correctly copied items and memory performance refer to count data with a maximum of 12.

Furthermore, a two-sample *t*-test showed that offloading behaviour in the choice sub-conditions did not differ between the conditions with and without announcement of the memory test. Hence, the participants in the choice/informed condition and the choice/uninformed condition did not differ in the openings of the model window, *t*(84) = −0.39, *p* = .693, $\eta_{p}^{2}<.01$, 95% CI = [0.00, 0.06], as well as the initially correctly copied items, *t*(84) = 0.78, *p* = .439, $\eta_{p}^{2}=.01$, 95% CI = [0.00, 0.08]. This finding contrasts with the observation of Experiment 2, in which memory test announcement under free-choice conditions resulted in reduced offloading behaviour. We will further elaborate on this in the “General discussion” section.

#### Immediate task performance

We exploratorily analysed trial duration (Each trial had to be solved correctly.) as a proxy for immediate task performance within the Pattern Copy Task. We observed a faster completion of the trials when participants were not aware of the upcoming memory test (uninformed condition) than when they expected the upcoming memory test (informed condition, see Table 8). A 2 × 2 exploratory between-subjects ANOVA with memory test announcement as well as offloading condition confirmed that this difference was significant. The participants in the uninformed condition solved the task faster than the participants in the informed condition, *F*(1, 168) = 8.54, *p* = .004, $\eta_{p}^{2}=.05$, 95% CI = [0.005, 0.12]. However, there was no main effect of the offloading condition (forced vs. choice), *F*(1, 168) = 2.70, *p* = .102, $\eta_{p}^{2}=.01$, 95% CI = [0.00, 0.07], as well as no interaction between both variables, *F*(1, 168) = 2.68, *p* = .103, $\eta_{p}^{2}=.01$, 95% CI = [0.00, 0.07].

#### Memory performance

**Figure 2.**
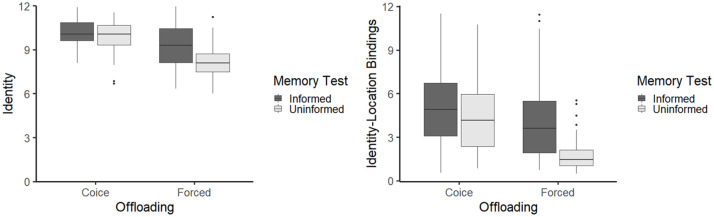
Interaction effect of the independent variables announcement of the memory test and the offloading condition on memory performance in Experiment 3.

In the conditions forcing participants to offload maximally, awareness of the upcoming memory test increased memory performance almost to the level of the condition with free-choice offloading behaviour (see Figure 2). For both proxies of memory performance, we conducted a separate 2 × 2 between-subject ANOVA with announcement of the memory test and offloading condition as the independent variables as well as memory performance as the dependent variable. We observed interactions between the independent variables for both proxies of memory performance “identity,” *F*(1, 168) = 6.89, *p* = .009, $\eta_{p}^{2}=.04$, 95% CI = [0.002, 0.11], as well as “identity-location bindings,” *F*(1, 168) = 5.99, *p* = .015, $\eta_{p}^{2}=.03$, 95% CI = [0.001, 0.10]. In addition, all main effects in both analyses reached significance, all *F*(1, 168)s ⩾ 13.92, all *p*s *<* .001, all $\eta_{p}^{2}s\geq .08$.

To further investigate the interaction effects, we conducted two-sample *t*-tests. With regard to both memory variables (identity and identity-location bindings), we observed less accurate memory performance in the forced/uninformed condition than in all other groups, all *t*(84)s ⩾ 4.17, all *p*s *<* .001, all $\eta_{p}^{2}s\geq .18$. Furthermore, we observed no difference in memory accuracy (identity and identity-location bindings) between the choice/informed and choice/uninformed conditions, all *t*(84)s ⩽ 1.04, all *p*s ⩾ .302, all $\eta_{p}^{2}s\leq .01$. Whether or not the forced/informed condition reached the level of the free-choice conditions differed between the two proxies of memory accuracy. When analysing only the identity of the recalled objects, the participants in the forced/informed condition showed a lower recognition accuracy than the participants in the choice/informed condition as well as the choice/uninformed condition, all *t*(84)s ⩾ 2.21, all *p*s ⩽ .029, all $\eta_{p}^{2}s\geq .05$. However, this difference was absent when analysing identity-location bindings. Here, the participants in the forced/informed condition did not differ in their subsequent memory performance from the participants in the choice/informed condition as well as the participants in the choice/uninformed condition, all *t*(84)s ⩽ 1.59, all *p*s ⩾ .115, all $\eta_{p}^{2}s\leq .03$.

In line with the previous two experiments, exploratory correlational analyses within the free-choice conditions revealed that an increased amount of cognitive offloading was associated with a lower subsequent memory performance in the choice conditions (see Table 9 as well as Figures S3.1 and S3.2 of the ESM). Due to the study design, there was hardly any variance in offloading behaviour in the forced condition. Therefore, we did not conduct correlational analyses for these groups. Furthermore, we did not repeat the mediation analyses of Experiments 1 and 2 as we directly manipulated offloading behaviour in this experiment.

**Table 9.** Pearson’s correlations between cognitive offloading and memory performance in Experiment 3.

	Identity	
	Choice	
	Uninformed	Informed
Openings of the model window	−.80***	−.55**
Initially correctly copied items	.70***	.57***
	Identity-location bindings	
	Choice	
	Uninformed	Informed
Openings of the model window	−.69***	−.38*
Initially correctly copied items	.78***	.60***

* *p* < .05, **p < .01, ***p < .001.

#### Working memory capacity

With regard to the Visual Pattern Test, 2 × 2 between-subjects ANOVAs with announcement of the memory test and offloading condition as the independent variables revealed no group differences or interactions, all *Fs*(1, 168) ⩽ 1.25, all *ps* ⩾ .265, all $\eta_{p}^{2}s<.01$, all BF_01_s ⩾ 3.38. With regard to the Corsi Blocks Task, the participants in the uninformed condition showed a lower working memory capacity for temporal sequences of spatial locations than the participants in the informed condition, *F*(1, 168) = 4.43, *p* = .039, $\eta_{p}^{2}=.02$, 95% CI = [0.00, 0.09], BF_01_ = 0.82. This matches the results of the Corsi Blocks Task in Experiment 2. As the Visual Patterns Test and Corsi Blocks Task were conducted after the experimental manipulation in the offloading task (to delay the memory test), it therefore seems likely that the experimental manipulation induced the reduced Corsi Blocks capacity. Offloading behaviour (in the free-choice conditions), however, was uncorrelated with capacity in the Corsi Blocks Task, all |*r*|s(41) ⩽ .23, all *p*s ⩾ .138. Furthermore, we observed no significant correlations between capacity in the Corsi Blocks Task and performance in the two memory measures all |*r*|s(41) ⩽ .28, all *p*s ⩾ .066 (exploratory analyses; see Table S3 in the ESM for all correlations). We observed no other group differences or interaction effects in the Corsi Blocks Task, all *F*s(1, 168) ⩽ 1.96, all *p*s ⩾ .163, all $\eta_{p}^{2}s<.01$, all BF_01_s ⩾ 2.04. Given the absence of group differences in the Visual Patterns Test (which is conceptually closer to the Pattern Copy Task) as well as no correlations of the capacity in the Corsi Blocks Task, we will not address this issue any further.

## General discussion

In the present research, we studied how offloading behaviour affects memory performance for the offloaded information. Our first two experiments demonstrate that cognitive offloading induces a trade-off between immediate task performance in the Pattern Copy Task and the formation of memory representations for the information presented in this task (within the same experiments and participants). In other words, while cognitive offloading accelerated task processing, it interfered with the formation of memory for the processed information. This finding replicates and extends previous results reported by Morgan et al. (2009; see also Morgan et al., 2013; Waldron et al., 2007) on longer retention intervals exceeding working memory maintenance as well as on stimuli depicting naturalistic objects (rather than coloured squares). This pattern of results appears to be genuine for an implicit formation of memory representations (i.e., Experiment 1 and the uninformed conditions of Experiment 2). Most importantly, the effect also arose when we directly manipulated offloading behaviour in Experiment 3. When participants were unaware of the memory test, their memory performance was more accurate when they were offloading less under free-choice conditions than when they were forced to offload to a maximum extent.

With regard to the explicit formation of memory representations, the results were a bit more mixed. In Experiment 2, participants who were aware of the upcoming memory test reduced their amount of cognitive offloading and subsequently revealed more accurate long-term memory performance. In the conditions with free choice in Experiment 3, however, we did not observe such a reduction of offloading behaviour induced by the awareness of the upcoming memory test relative to the condition without such awareness. This discrepancy raises questions regarding the generality, reliability, or magnitude of the effect of test announcement on offloading behaviour. We will therefore only briefly discuss this finding below without drawing strong conclusions from it. Nevertheless, what remained consistent in Experiments 2 and 3 is that the amount of offloading inversely matched the subsequent memory performance under free choice and informed conditions. Whereas the reduction of offloading in the informed conditions in Experiment 2 came along with more accurate subsequent memory, the absence of such a reduction in Experiment 3 was accompanied by the absence of differences in the subsequent memory tests. This consistency seems to suggest that there is a link between offloading and memory performance. Such a link would suggest that cognitive resources which remain “free” due to offloading (i.e., unused working memory capacity) are “lost” and do not contribute to the formation of memory. However, the forced offloading conditions in Experiment 3 prove the strong version of this assumption to be wrong.

The results of Experiment 3 demonstrate that the amount of offloading does not necessarily determine memory accuracy. On the contrary, despite being forced to offload maximally, the participants in the forced/informed condition of this experiment showed almost the same memory accuracy as those participants in the choice/informed and choice/uninformed condition. Therefore, at least under the extreme conditions of enforced maximum offloading, it seems possible to counteract the negative impact of offloading on the formation of memory—potentially through the successful use of released cognitive resources to form a strong long-term memory. What remains an open question for future research is why such beneficial effects of released resources do not arise with free-choice offloading behaviour. There are at least two speculative explanations for this pattern. First, it is possible that it is costly in terms of mental resources to coordinate a task solution at a medium memory load with a simultaneous medium use of released resources. Second, it could be that a minimum amount of released resources is necessary to reveal their positive effects. In the case of forced offloading, there are probably more “free” than “used” resources which might have enabled their impact on the general pattern of results.

A remarkable finding across all experiments is the high correlations between offloading behaviour and memory performance on an individual level. These correlations were present within implicit (i.e., uninformed) as well as explicit (i.e., informed) set-ups of the experiments and support our findings on the group level in Experiments 1 and 2. More pronounced offloading behaviour diminishes subsequent memory performance for the offloaded information. It seems important to note, however, that this correlational relationship is not deterministic. In the third experiment, we forced participants to offload to a maximum extent and observed differences on the group level in the memory performance despite constant offloading behaviour on the individual level.

A central question for the interpretation of our results is whether offloading behaviour itself impacts memory performance. Alternatively, the participants in the lockout conditions could have used the 2-s lockout times to rehearse the visual information and thus showed an improved subsequent memory performance relative to participants in the no lockout conditions, independent of offloading behaviour. Contrary to this view, however, our mediation analyses highlight the detrimental effects of offloading for the formation of memory representations. The impact of our manipulations (lockout/no lockout and informed/uninformed memory test) on subsequent memory performance was mediated by offloading behaviour. This suggests that offloading behaviour itself is associated with memory accuracy.

### The trade-off of cognitive offloading

Beyond the conceptual replication of previous reports of a trade-off between immediate positive and subsequent negative effects of offloading memory processes in the Pattern Copy Task (Morgan et al., 2009, 2013; Waldron et al., 2007), our results also converge with studies reporting the detrimental effects of cognitive offloading in other paradigms (Eskritt & Ma, 2014; Kelly & Risko, 2019; O’Hara & Payne, 1998; Pyke & LeFevre, 2011; Sparrow et al., 2011). Thus, the consequences of cognitive offloading are comparable across different offloading paradigms. However, individual differences in offloading behaviour appear to be uncorrelated across different research paradigms (Meyerhoff et al., preprint), which urges for further research investigating the relationship of consequences of offloading behaviour across paradigms. Returning to the converging findings with regard to the consequences of cognitive offloading, Pyke and LeFevre (2011) observed that using a calculator in an alphanumerical test led to a higher response accuracy but in return to a worse subsequent recall of the solution. They therefore concluded that using a calculator results in less active learning than self-generating answers. With regard to our Pattern Copy Task, temporarily high loads of working memory (i.e., copying multiple objects simultaneously) rather than continuously low loads (i.e., copying the objects sequentially) enhanced subsequent memory accuracy under free-choice conditions, although all participants had to handle the same overall amount of information.

Please note that these temporarily high loads of working memory enhanced memory for the identity of the objects as well as their locations relative to each other. The general pattern of our findings matches the notion of desirable difficulties. Conceptually, desirable difficulties are supposed to enforce a more effortful and therefore more elaborate processing of information to enhance long-term learning (Bjork & Bjork, 2011). Thus, saving a lot of information internally places a high effort on internal cognitive resources, which in return might lead to a deep processing of the information at hand and foster learning. Within this account, avoiding offloading behaviour (such as in Experiment 2) to foster long-term learning could be seen as self-generated desirable difficulty. Nevertheless, as differences in memory could also arise with the same amount of offloading (as observed in Experiment 3), it remains possible that participants could use released cognitive resources to acquire more accurate memory representations. Whether these released resources are employed in a manner that would be consistent with a self-generated desirable difficulty is one of the open questions to pursue in future research.

Our findings are also compatible with the framework of Salomon (1990), who proposed effects *with* technology on task processing as well as effects *of* technology on the development of cognitive abilities in the field of learning sciences. Effects *with* technology are supposed to affect immediate task processing due to utilising technical tools, whereas effects *of* technology are potential long-term consequences (e.g., development of cognitive abilities) caused by preceding interactions with technology. Hence, cognitive offloading with technical tools might enable performance beyond internal cognitive limitations and thus increase immediate performance (Kirsh & Maglio, 1994; Salomon & Perkins, 2005). However, what users learn in this context appears to be how to effectively utilise the offloading device rather than solving the problem at hand with one’s own cognitive abilities (Moritz et al., 2020). In return, it might be the absence of practice and routine in using internal resources which causes the detrimental effects of cognitive offloading (Risko & Gilbert, 2016; Salomon, 1990). Recent findings revealed that already children selectively use tools to overcome cognitive limitations (Armitage et al., 2020; Bulley et al., 2020). Therefore, effects *with* and *of* technology might already constitute an important trade-off to consider at young ages.

### Positive consequences of cognitive offloading

Although Experiments 1 and 2, as well as the uninformed conditions in Experiment 3, constantly suggest that cognitive offloading is harmful for the formation of memory, one condition in our third experiment demonstrates that participants could counteract these detrimental effects under certain circumstances. If it was necessary to acquire memory representations and there was no possibility to regulate offloading behaviour, the participants in the forced/informed condition might have been able to use released internal resources to improve memory performance. Please note that this improvement is relative to a condition in which participants are not aware of the subsequent memory test and therefore have no incentive to memorise the information. Overall, this improvement brings these participants (almost) back to the level of the participants who were able to freely choose their offloading behaviour. As there is no benefit above the level of freely chosen offloading behaviour, this might also be a reason why the participants in our Experiment 2 solved that task with more internal memory rather than with more offloading when they were aware of the memory test.

Our observation that participants in principle might be capable of taking advantage of released resources matches a common argument in favour of cognitive offloading. One might consider that such released resources which come along with externalisations (Kirsh, 2010) could serve a deeper elaboration of the processed information (Sweller et al., 1998). In return, such a deeper elaboration could cause stronger memory representations (Craik & Lockhart, 1972). From the results of our experiments, it appears that such an argument rests on two essential preconditions. First, participants need to have the goal to foster long-term learning, as detrimental consequences under implicit learning conditions are likely. Second, the amount of released cognitive resources needs to be substantially large to contribute to learning. In our experiments, we only observed such a contribution of released resources when we forced participants to complete the task with a minimum of internal resources.

A central objective for future research is to disentangle potentially beneficial effects of freed cognitive resources from potentially beneficial effects of additional time. Descriptively, the participants took longer in the forced/informed condition than the forced/uninformed condition to perform a trial in the Pattern Copy Task. Thus, it remains possible that at least a part of the improved memory performance in this condition stems from the additional time rather than the successful use of released cognitive resources. Nevertheless, it appears unlikely that the additional time alone explains our pattern of results as offloading behaviour rather than additional rehearsal time was the critical predictor for memory performance in Experiments 1 and 2.

It seems noteworthy that the announcement of upcoming memory tests does not generally result in beneficial effects of cognitive offloading. In an experiment reported by Sparrow et al. (2011), the participants transferred trivia statements into a computer document. Whereas the participants remembered fewer of these statements when they believed their document was saved in the computer rather than when they believed their document was erased, announcing the memory test had no effect on performance. Given the substantial differences between both paradigms (task, materials, difficulty, etc.), it seems hardly helpful to speculate about the origin of the differences in the results. Nevertheless, this discrepancy again shows that the interactions between the released resources due to cognitive offloading and the goal of acquiring new mental representations is understudied and not well understood yet. Given the widespread distribution of modern technical tools that allow for cognitive offloading, however, a deeper understanding of this interaction is highly relevant to enable an appropriate usage of such tools.

Our experiments as well as those of Sparrow et al. (2011) focused on the interplay of offloading and memory accuracy for the offloaded information itself. Beyond the offloaded information itself, however, cognitive offloading could also affect cognitive performance for unrelated materials or in unrelated tasks (Runge et al., 2019; Storm & Stone, 2015). For instance, Storm and Stone (2015) observed that saving information in a technical tool before studying further information improved the memory performance of the latter information (i.e., reduced interference from the first information on the second). Furthermore, there appear to be carry-over effects in offloading behaviour between successive tasks (i.e., participants relying more on offloading in one task also rely more on offloading in a subsequent task; Storm et al., 2016).^10^

### Cognitive offloading as a strategy

Given the growing impact on people’s everyday lives by technical tools, including external memories (Finley et al., 2018), a careful consideration of the apparent benefits and the hidden risks of cognitive offloading seems needed to avoid unintended detrimental long-term effects. First, it appears necessary to evaluate the goal of the task at hand. If the goal focuses on immediate performance, our study suggests that the adequate strategy would be increasing externalisations. In contrast, if the task’s goal involves components of memorisation or learning, different strategies should be applied. On one hand, offloading behaviour could be avoided to create desirable difficulties and foster learning. On the other hand—if avoiding offloading is not possible—released resources due to offloading might be activated to foster learning.

In Experiment 2, we observed that participants who were aware of the upcoming memory test offloaded less but had a better memory than participants who were not aware of the upcoming memory test. This finding suggests that the participants might have been aware of the negative consequences of cognitive offloading or at least did not believe that cognitive offloading could be used beneficially, so that they decided to rely more on their internal memory than on externalisations. Critically, we did not observe such a change in offloading behaviour based on the announcement of the memory test in Experiment 3 (choice condition). Therefore, from our experiments, we cannot conclude whether participants do or do not have metacognitive knowledge about the impact of cognitive offloading on memory. Related studies investigating metacognitions in the context of cognitive offloading, however, showed that metacognitive beliefs about one’s own cognition explain at least some individual differences in offloading behaviour (e.g., Gilbert et al., 2020; Hu et al., 2019 but see Grinschgl, Meyerhoff, Schwan, & Papenmeier, 2020). Nevertheless, the present findings urge for further research directly investigating how metacognitions about the impact of offloading (rather than metacognitions about one’s own cognition as investigated by previous studies) alter offloading behaviour across tasks with varying goals focusing either on immediate performance or subsequent memory. A plausible venue for such further research could be to investigate how manipulations of metacognitions impact strategy selection in offloading tasks as well as memory performance.

## Conclusion

Taken together, we can derive the following insights from our experiments: First, there is a trade-off between benefits and risks of cognitive offloading. Cognitive offloading increases immediate task performance but also diminishes subsequent memory performance for the offloaded information. Second, under free-choice offloading, more cognitive offloading was associated with a lower subsequent memory performance on the group level as well as on the level of individual differences. Third, announcing subsequent testing could compensate for at least some of the detrimental effects of cognitive offloading on memory acquisition. Fourth, reducing the amount of offloading as a self-generated desirable difficulty as well as a taking advantage of released cognitive resources might reflect competing strategies when counteracting the detrimental effects of cognitive offloading. Fifth and finally, resources released by cognitive offloading only contribute to the formation of memories in explicit learning contexts (i.e., when participants have the goal to learn) but are rather “lost” without such a learning context.

## Supplemental Material

sj-docx-1-qjp-10.1177_17470218211008060 – Supplemental material for Consequences of cognitive offloading: Boosting performance but diminishing memory Click here for additional data file. Supplemental material, sj-docx-1-qjp-10.1177_17470218211008060 for Consequences of cognitive offloading: Boosting performance but diminishing memory by Sandra Grinschgl, Frank Papenmeier and Hauke S Meyerhoff in Quarterly Journal of Experimental Psychology

## Notes

1. We used G*Power (Faul et al., 2007) to calculate the required sample size for a 2 × 2 between-subjects analysis of variance (ANOVA; i.e., the main statistical test for Experiments 2 and 3). The calculation suggests a total sample size of 171 participants. To equally distribute the participants across the conditions, we tested 172 participants.

2. Exploratory analyses showed that all the reported results and conclusions remained equivalent when participants who reported a priori test awareness were included in the analyses (*N* = 186).

3. For these exploratory analyses, we report a rather large number of tests without adjustments of the significance level. Please note, however, that we (1) treat the results as exploratory, (2) almost all corresponding *p*-values are below .001 so that even conservative corrections would leave the results unaffected, and (3) we only interpret results which emerge consistently across all three experiments.

4. The Bayesian independent *t*-tests were performed with a prior described by the Cauchy distribution centred around 0 and with a width parameter of 0.707. All Bayesian analyses were performed with JASP (JASP Team, 2019; jasp-stats.org). BF_01_ refers to evidence for the null hypothesis relative to the alternative hypothesis.

5. When exploratorily including participants that were excluded due to false memory test awareness in the analysis (*N* = 191), the results remain essentially the same and therefore do not affect our interpretation.

6. Please note: We conducted Experiment 2 before Experiments 1 and 3. As participants performed rather well on the memory test in this experiment, we increased the number of images for Experiments 1 and 3.

7. The Bayesian ANOVAs were performed with the default prior of JASP (*r* scale fixed effects = 0.5).

8. Including these participants in the analysis (*N* = 199) turned the interactions between announcement of the memory test and offloading conditions from statistical significance into numerical trends (i.e., .05 < *p* < .1). However, as participants in the forced offloading condition still benefitted from test awareness with regard to their memory performance, such a change would not have a major impact on the interpretation of the current study. Furthermore, the group difference in the Corsi Blocks Task attenuates from statistical significance into a numerical trend when including participants with memory test awareness which deviated from their instructions.

9. Due to the forced offloading condition in this experiment, the proxies for offloading were of minor relevance. Nevertheless, we preregistered to analyse the number of openings of the model window and the number of initially correctly copied items to demonstrate that participants were offloading more when they were forced to do so than under free-choice conditions.

10. For the interested readers, we added plots displaying offloading behaviour across all trials and for each group within the Pattern Copy Task in the electronic supplement material (ESM; Figures S4, S5, and S6). These plots show that participants mostly maintained their level of offloading after the first few trials for the rest of the task.
