# Supplementary material for: Consequences of cognitive offloading: Boosting performance but diminishing memory
Source: Q J Exp Psychol (Hove). 2021 Apr 4;74(9):1477–96. doi: 10.1177/17470218211008060 (PMC8358584; doi:10.1177/17470218211008060)
Supplement: sj-docx-1-qjp-10.1177_17470218211008060 – Supplemental material for Consequences of cognitive offloading: Boosting performance but diminishing memory [file sj-docx-1-qjp-10.1177_17470218211008060.docx]

Supplementary Material for:

**Consequences of Cognitive Offloading: Boosting Performance But Diminishing Memory**

Sandra Grinschgl, Frank Papenmeier, Hauke S. Meyerhoff

**Electronic Supplementary Material**

**Scatter Plots for Experiment 1**


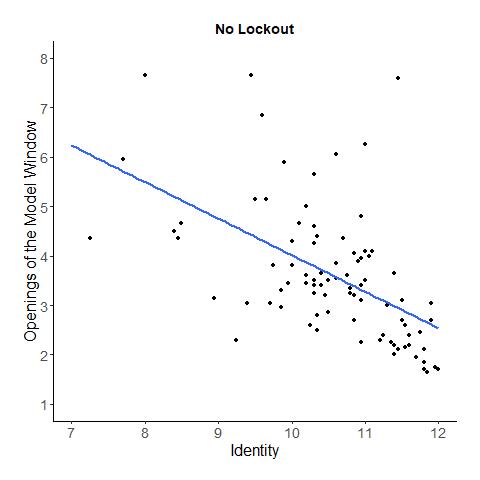

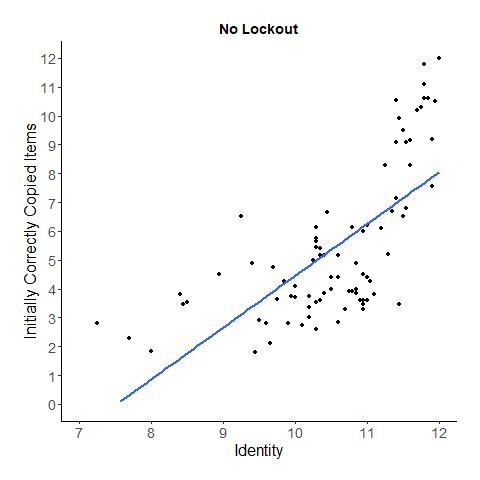

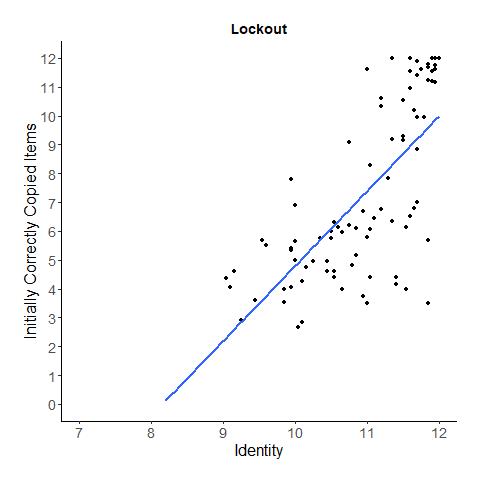

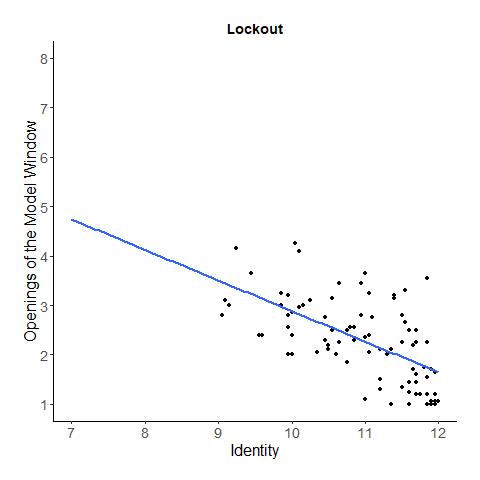

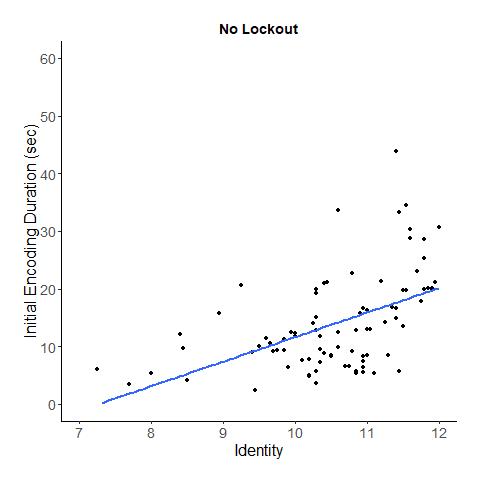

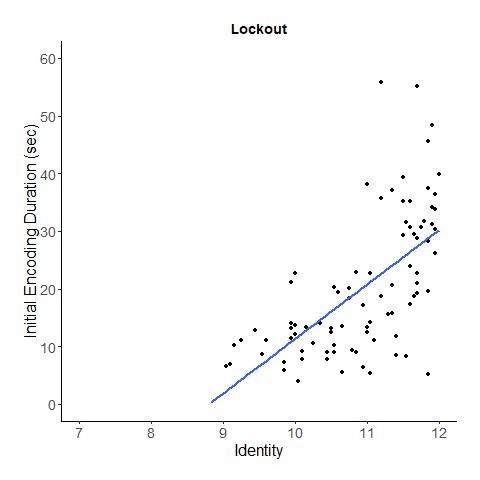


*Figure 1.1* Scatter Plots of Correlational Analyses between Cognitive Offloading and Memory Performance (Identity) for Experiment 1 (see also Table 2 in article).


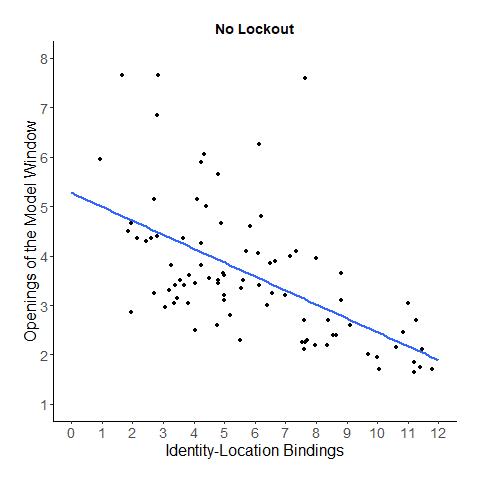

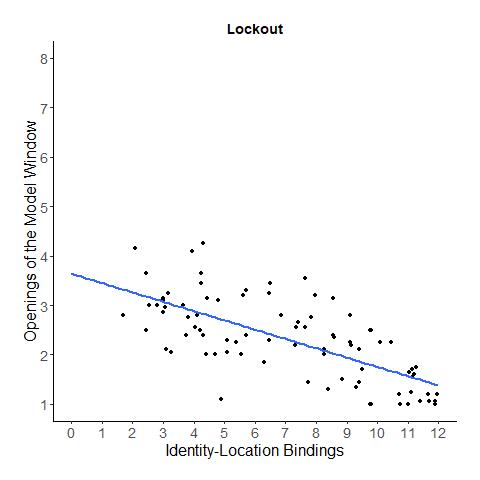

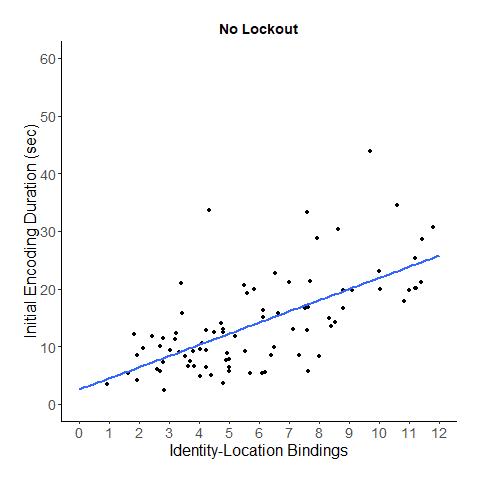

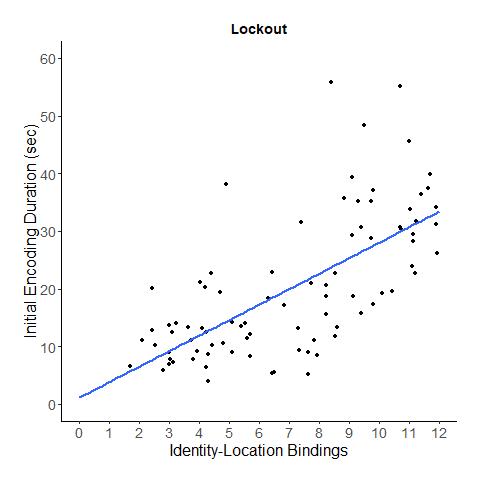

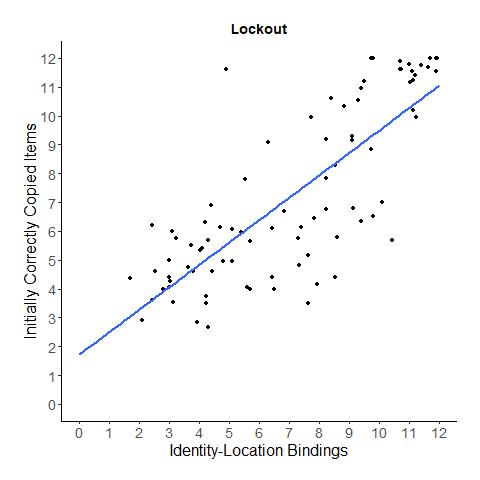

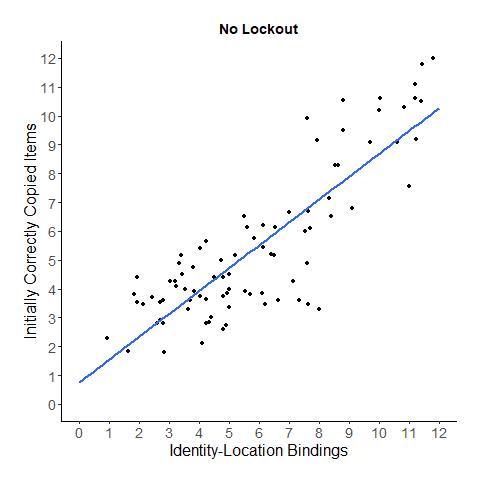


*Figure 1.2* Scatter Plots of Correlational Analyses between Cognitive Offloading and Memory Performance (Identity-Location Bindings) for Experiment 1 (see also Table 2 in article).


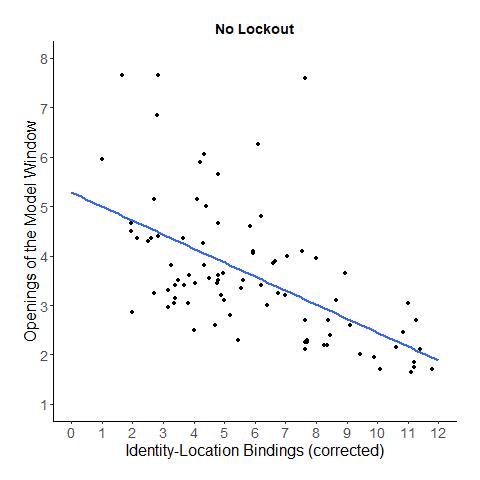

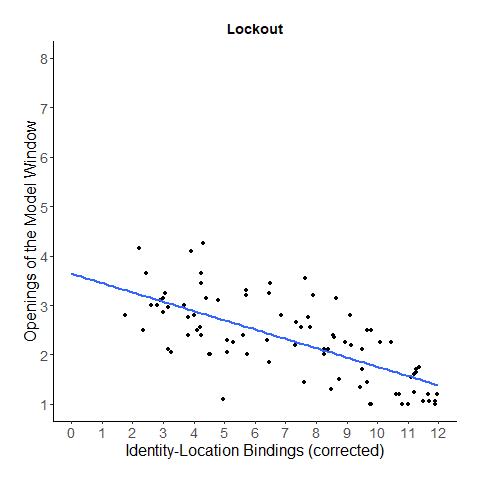

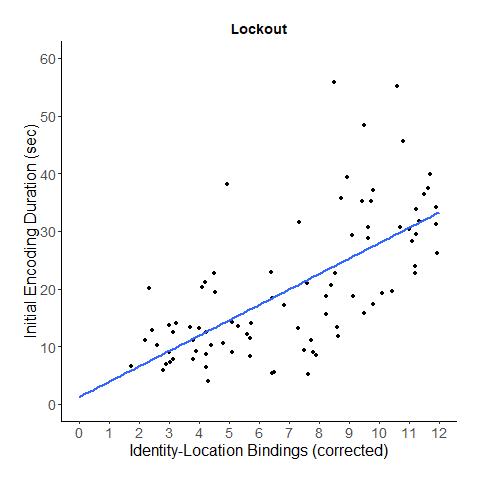

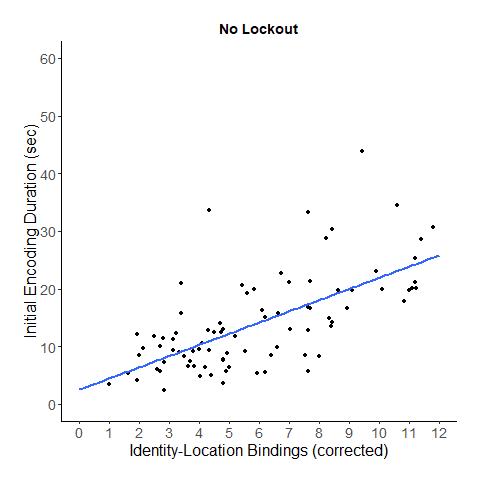

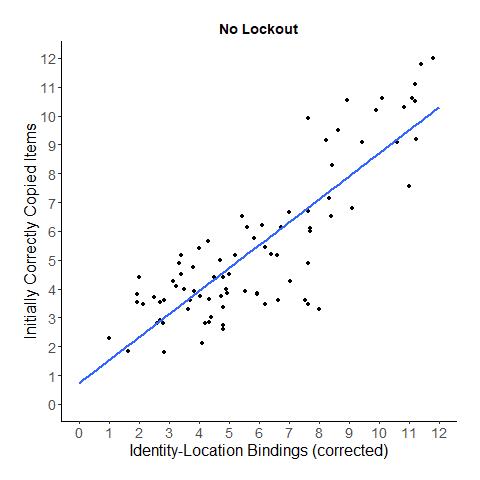

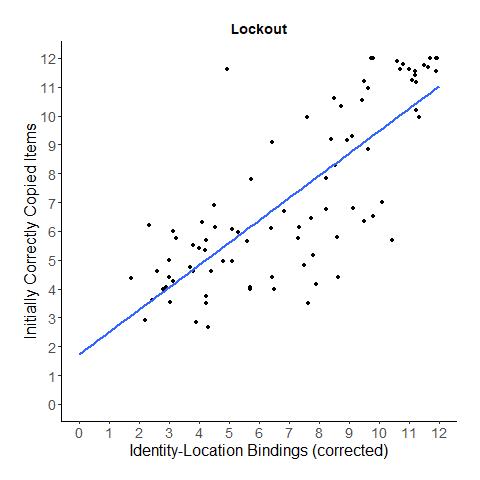


*Figure 1.3* Scatter Plots of Correlational Analyses between Cognitive Offloading and Memory Performance (Identity-Location Bindings corrected) for Experiment 1 (see Table 2 in article).

**Scatter Plots for Experiment 2**


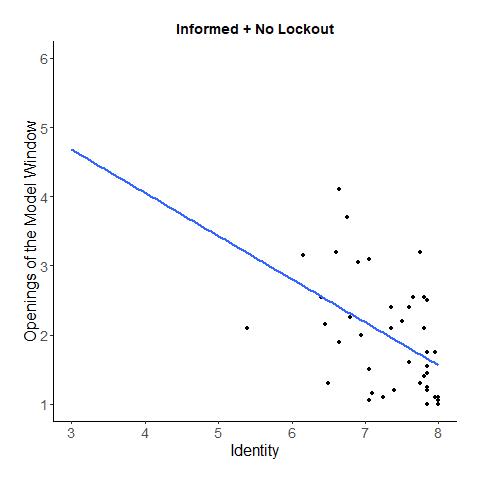

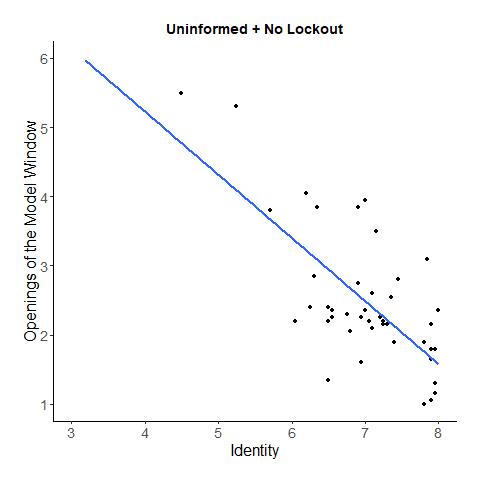

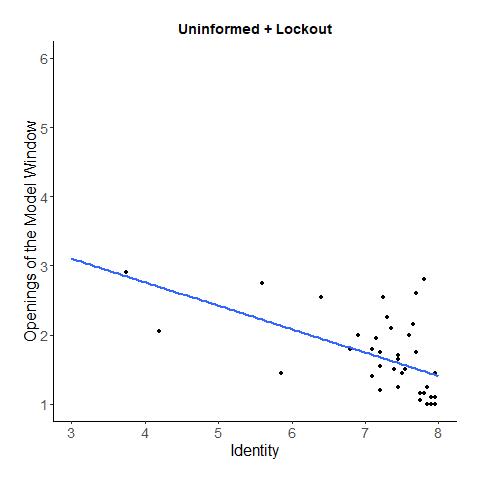

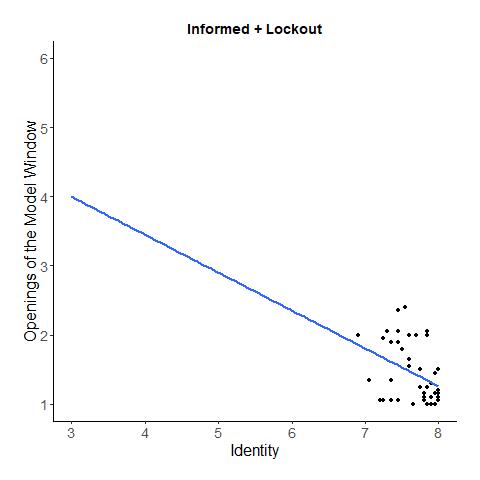

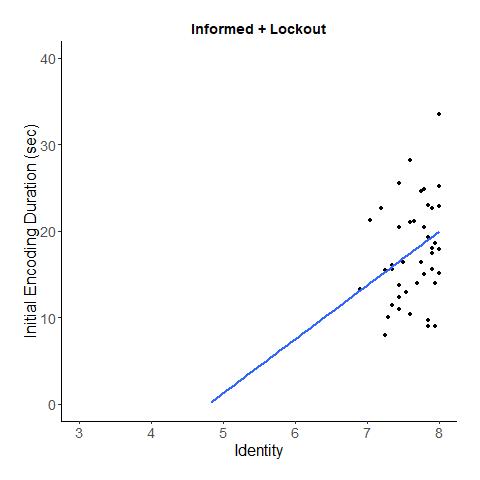

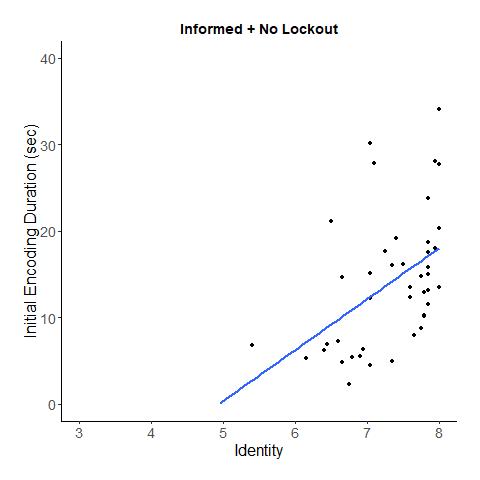

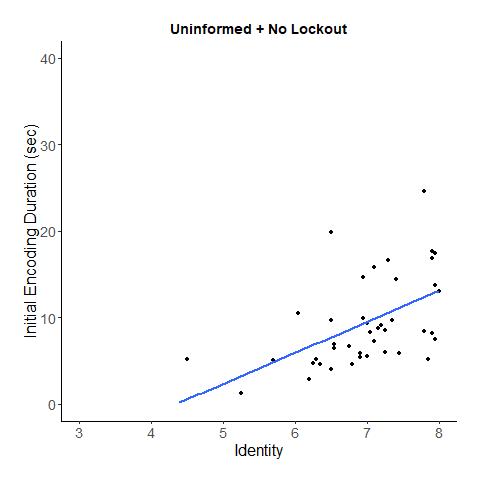

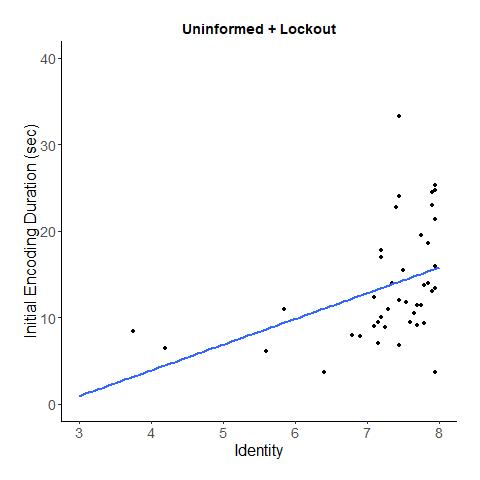

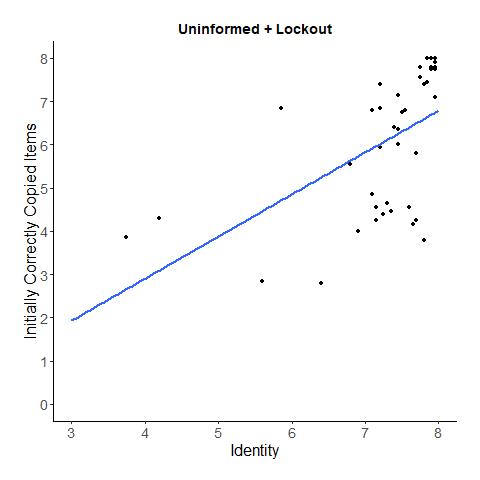

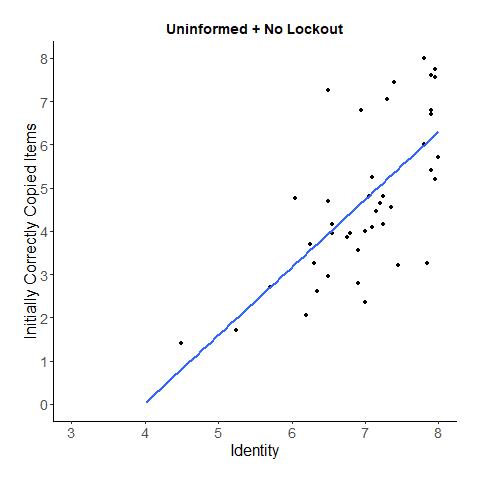

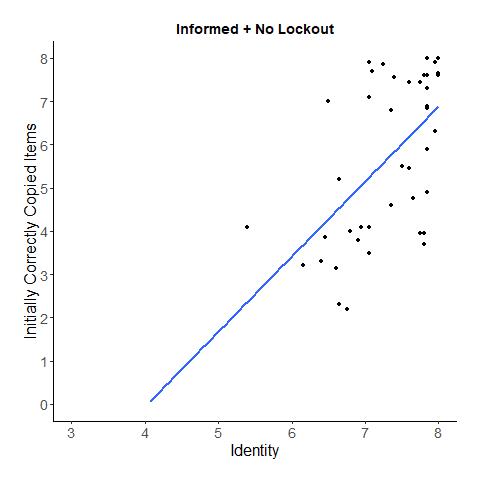

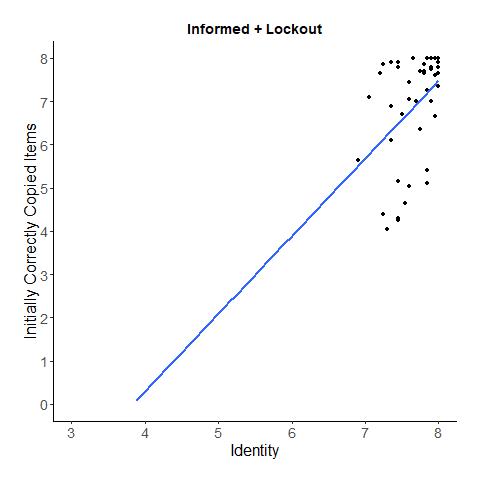


*Figure 2.1* Scatter Plots of Correlational Analyses between Cognitive Offloading and Memory Performance (Identity) for Experiment 2 (see also Table 5 in article).


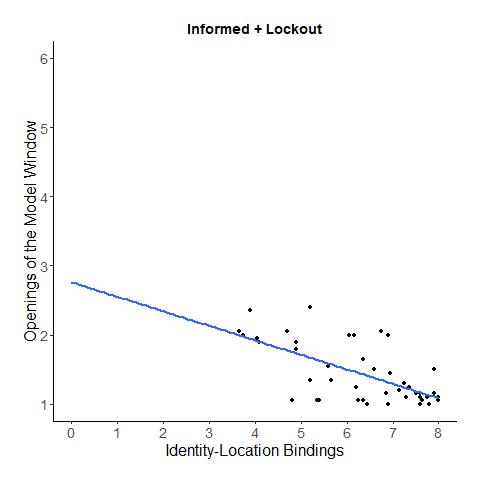

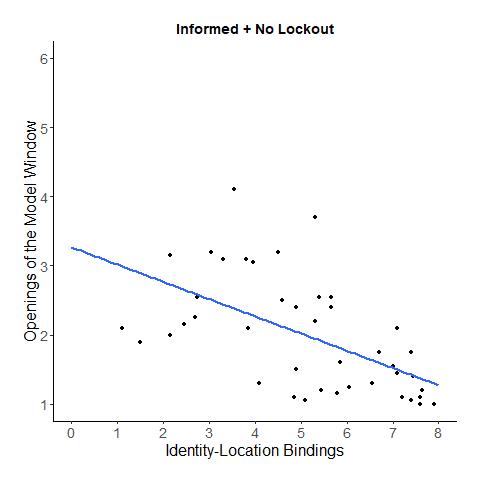

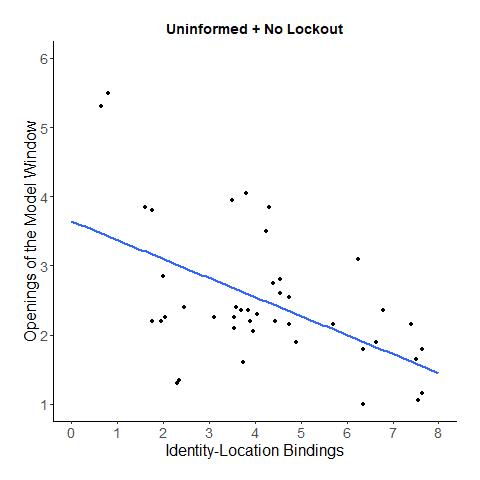

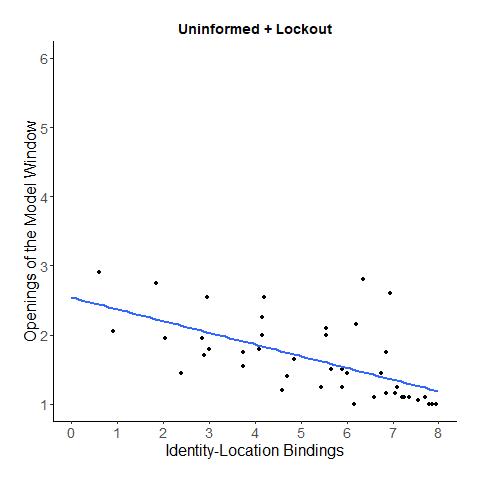

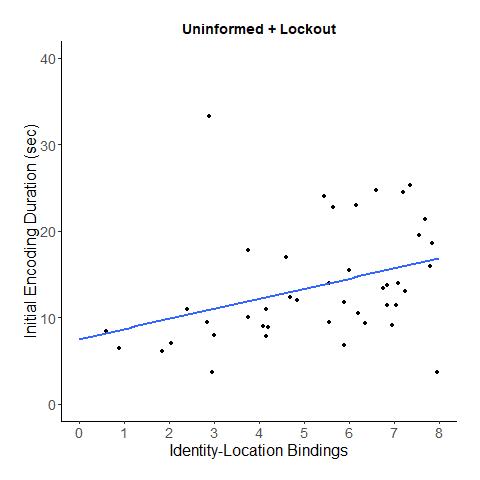

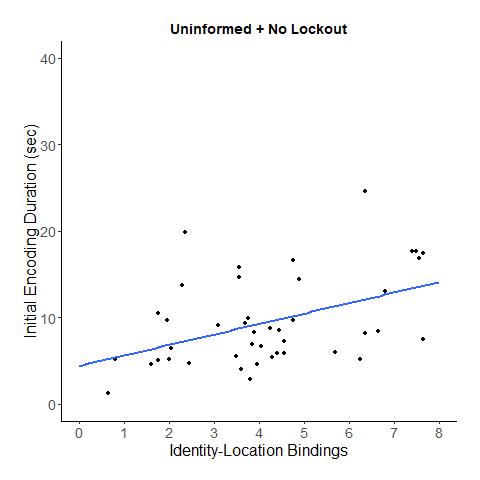

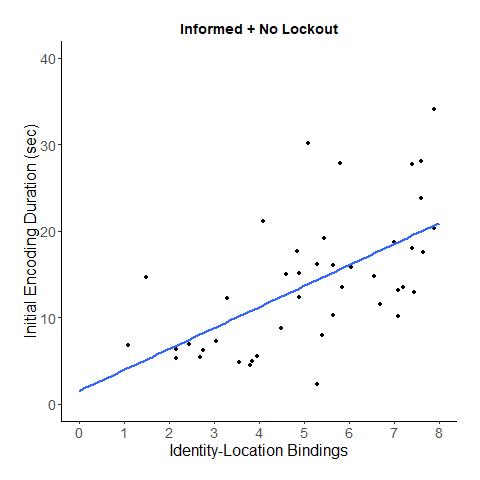

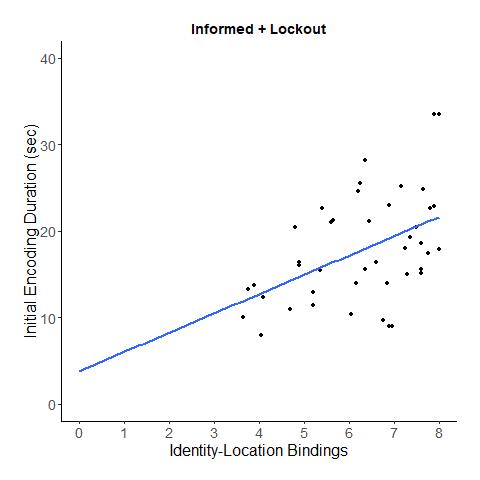

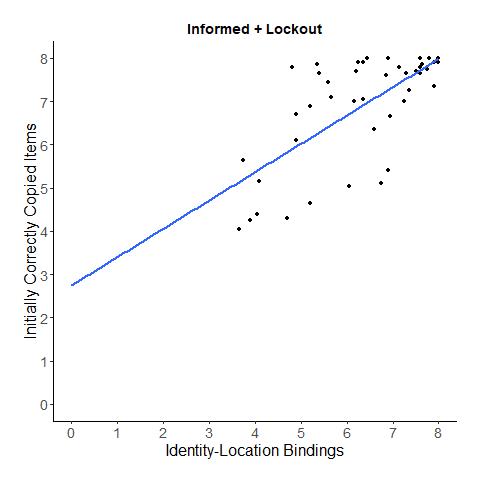

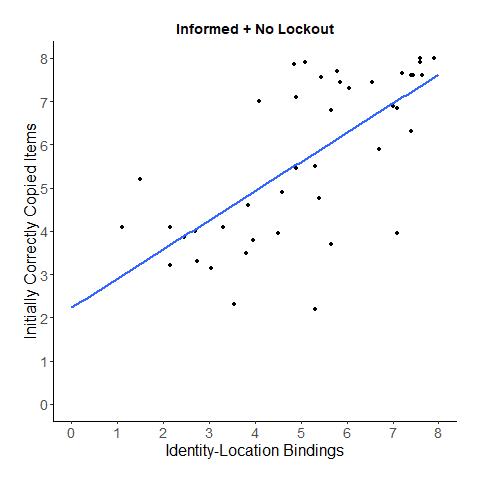

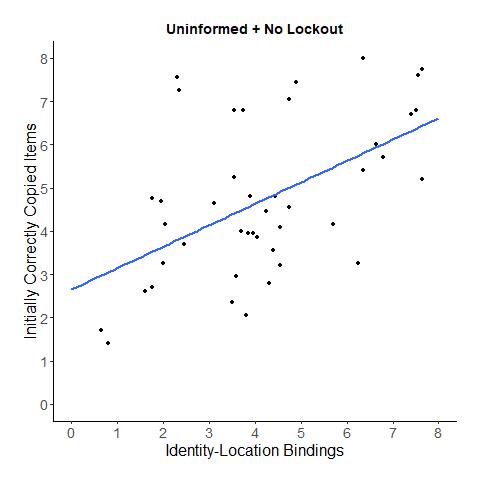

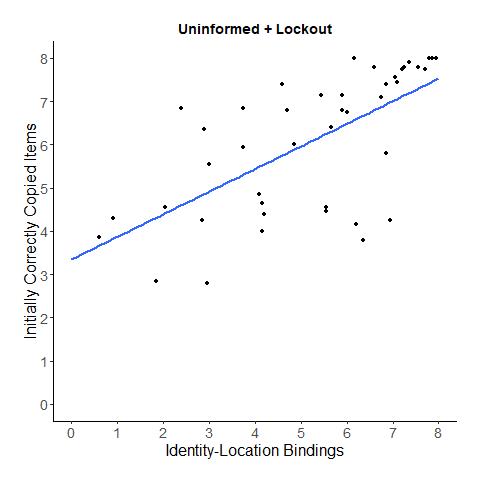


*Figure 2.2* Scatter Plots of Correlational Analyses between Cognitive Offloading and Memory Performance (Identity-Location Bindings) for Experiment 2 (see also Table 5 in article).

**Scatter Plots for Experiment 3**


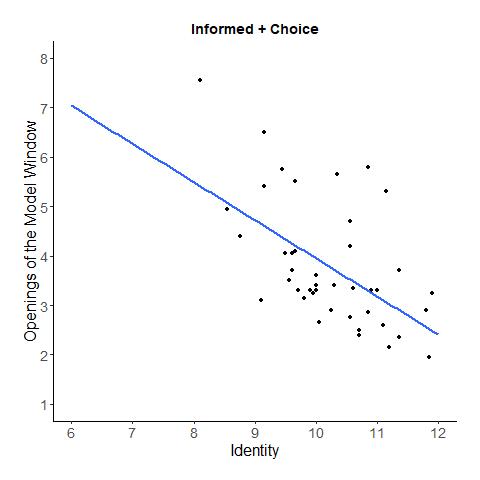

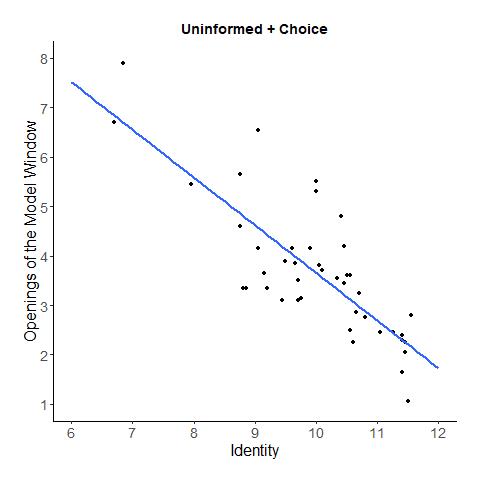

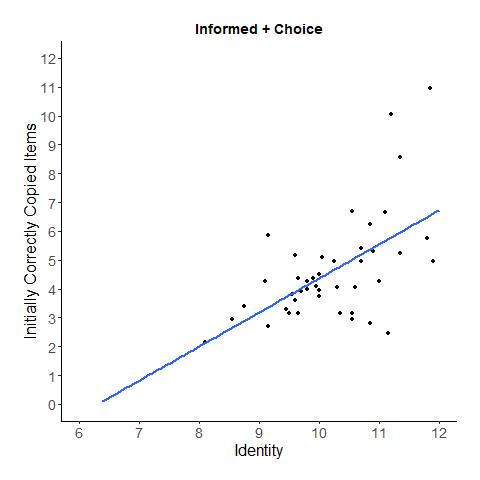

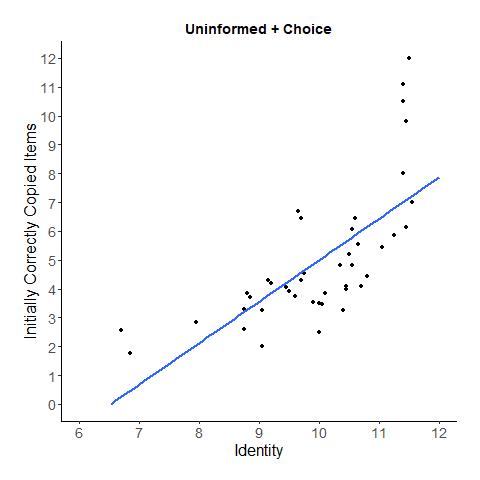


*Figure 3.1* Scatter Plots of Correlational Analyses between Cognitive Offloading and Memory Performance (Identity) for Experiment 3 (see also Table 9 in article).


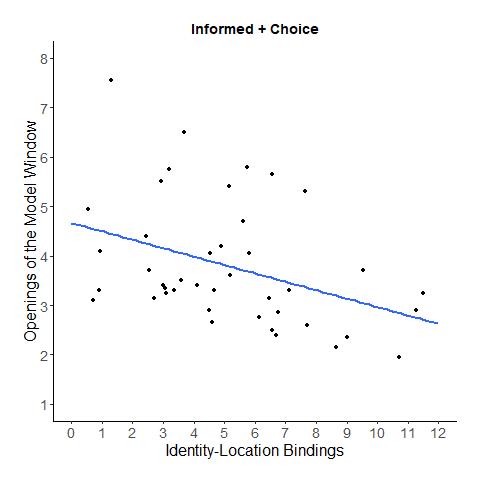

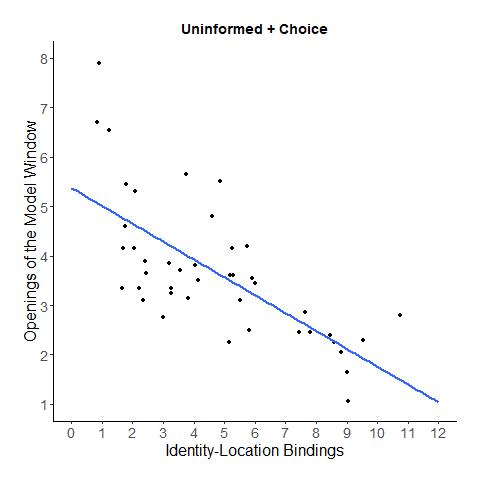

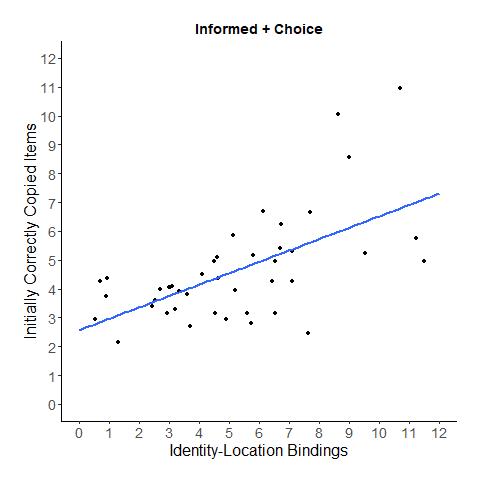

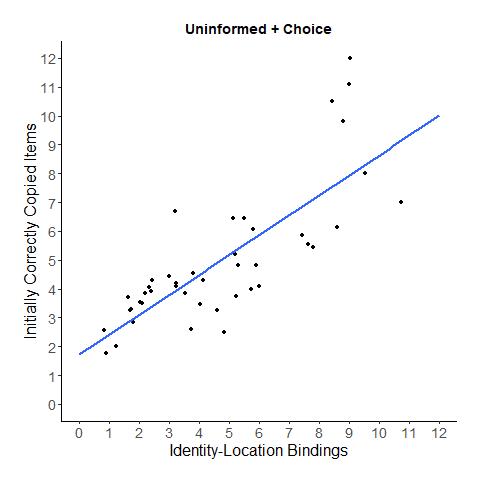


*Figure 3.2* Scatter Plots of Correlational Analyses between Cognitive Offloading and Memory Performance (Identity-Location Bindings) for Experiment 3 (see also Table 9 in article).

**Plots for Experiment 1**


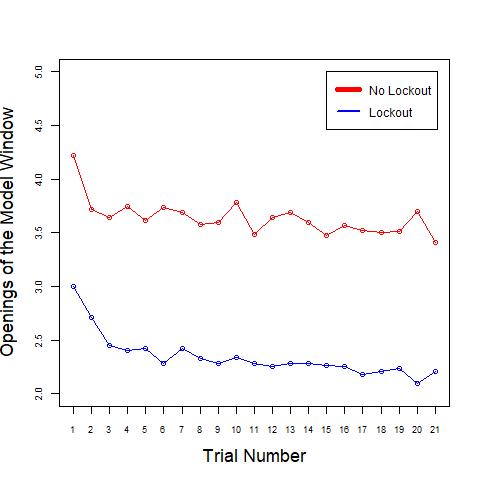

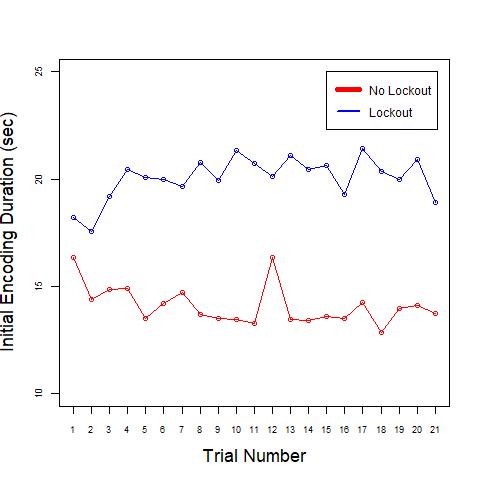

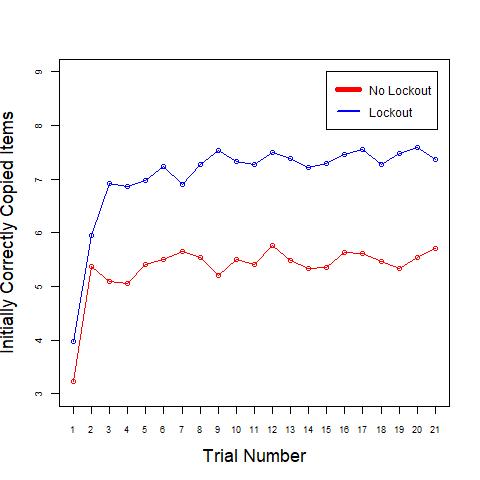


*Figure 4.* Plots of Offloading Behavior across Trials in Pattern Copy Task in Experiment 1. The first trial (Trial Number 1) was the practice trial, followed by twenty test trials (Trial Number 2-21).

**Plots for Experiment 2**


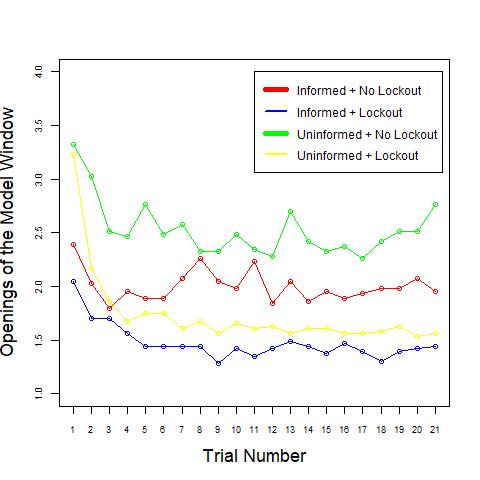

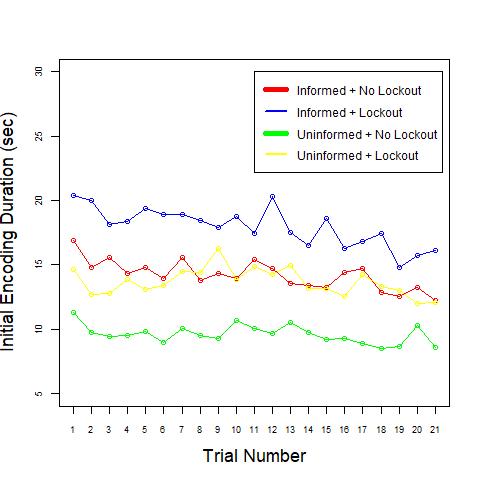

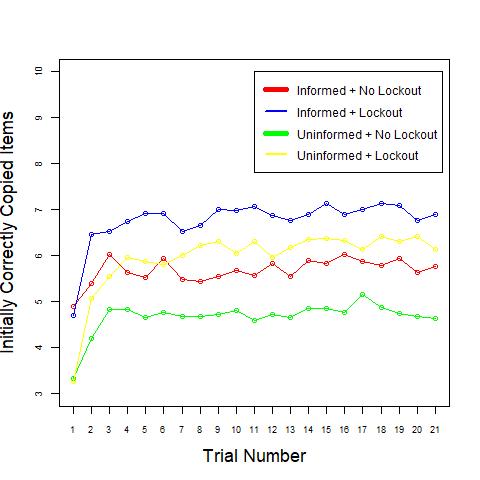


*Figure 5.* Plots of Offloading Behavior across Trials in Pattern Copy Task in Experiment 2. The first trial (Trial Number 1) was the practice trial, followed by twenty test trials (Trial Number 2-21).


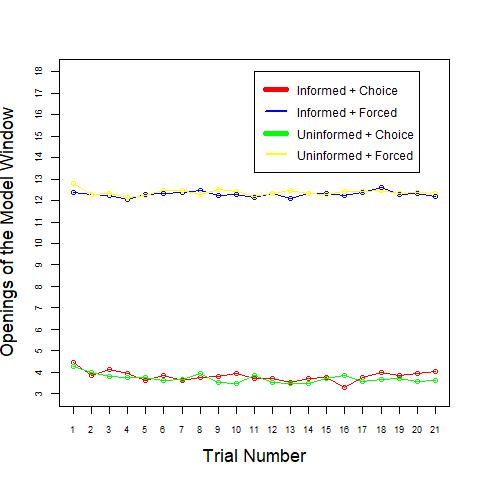
**Plots for Experiment 3**


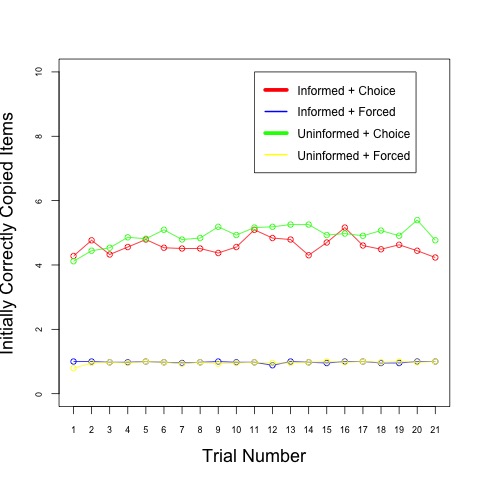


*Figure 6.* Plots of Offloading Behavior across Trials in Pattern Copy Task in Experiment 3. The first trial (Trial Number 1) was the practice trial, followed by twenty test trials (Trial Number 2-21).

**Correlations for Experiment 1**

Table 1

*Pearson-Correlations between Working Memory Capacity and Cognitive Offloading, Immediate Task Performance as well as Memory Performance in Experiment 1*

|  | **Cognitive Offloading**  Openings of the Model Window | |
| --- | --- | --- |
|  | No Lockout | Lockout |
| **Working Memory Capacity**  Visual Patterns Test  Corsi Blocks Task | -.35***  -.29** | -.22*  -.06 |
|  | Initial Encoding Duration (sec) | |
|  | No Lockout | Lockout |
| Visual Patterns Test  Corsi Blocks Task | .34**  .28** | -.04  -.02 |
|  | Initially Correctly Copied Items | |
|  | No Lockout | Lockout |
| Visual Patterns Test  Corsi Blocks Task | .39***  .38*** | -.11  .004 |
|  | **Immediate Task Performance**  Trial Duration (sec) | |
|  | No Lockout | Lockout |
| Visual Patterns Test  Corsi Blocks Task | .07  -.09 | -.32**  -.23* |
|  | Errors | |
|  | No Lockout | Lockout |
| Visual Patterns Test  Corsi Blocks Task | -.26*  -.09 | -.26*  -.04 |
|  | **Memory Performance**  Identity | |
|  | No Lockout | Lockout |
| Visual Patterns Test  Corsi Blocks Task | .28**  .26* | .09  -.13 |
|  | Identity-Location Bindings | |
|  | No Lockout | Lockout |
| Visual Patterns Test  Corsi Blocks Task | .38***  .33** | .17  -.08 |
|  | Identity-Location Bindings (corrected) | |
|  | No Lockout | Lockout |
| Visual Patterns Test  Corsi Blocks Task | .37***  .33** | .17  -.08 |

*Note:* * *p* < .05, ** *p* < .01, *** *p* < .001

**Correlations for Experiment 2**

Table 2

*Pearson-Correlations between Working Memory Capacity and Cognitive Offloading, Immediate Task Performance as well as Memory Performance in Experiment 2*

|  | **Cognitive Offloading**  Openings of the Model Window | | | |
| --- | --- | --- | --- | --- |
|  | No Lockout | | Lockout | |
|  | Uninformed | Informed | Uninformed | Informed |
| **Working Memory Capacity**  Visual Patterns Test  Corsi Blocks Task | -.51***  -.21 | -.32*  -.07 | -.38*  -.11 | -.33*  -.19 |
|  | Initial Encoding Duration (sec) | | | |
|  | No Lockout | | Lockout | |
|  | Uninformed | Informed | Uninformed | Informed |
| Visual Patterns Test  Corsi Blocks Task | .25  -.15 | .10  -.04 | .24  .15 | -.08  .005 |
|  | Initially Correctly Copied Items | | | |
|  | No Lockout | | Lockout | |
|  | Uninformed | Informed | Uninformed | Informed |
| Visual Patterns Test  Corsi Blocks Task | .38*  .01 | .34*  .14 | .24  .03 | -.21  .16 |
|  | **Immediate Task Performance**  Trial Duration (sec) | | | |
|  | No Lockout | | Lockout | |
|  | Uninformed | Informed | Uninformed | Informed |
| Visual Patterns Test  Corsi Blocks Task | -.27  -.41* | -.10  -.19 | -.01  -.08 | -.41**  -.18 |
|  | **Memory Performance**  Identity | | | |
|  | No Lockout | | Lockout | |
|  | Uninformed | Informed | Uninformed | Informed |
| Visual Patterns Test  Corsi Blocks Task | .37*  -.04 | .23  -.09 | .15  .21 | .04  .02 |
|  | Identity-Location Bindings | | | |
|  | No Lockout | | Lockout | |
|  | Uninformed | Informed | Uninformed | Informed |
| Visual Patterns Test  Corsi Blocks Task | .32*  .01 | .27  -.06 | .08  .09 | .19  .17 |

*Note:* * *p* < .05, ** *p* < .01, *** *p* < .001

**Correlations for Experiment 3**

Table 3

*Pearson-Correlations between Working Memory Capacity and Cognitive Offloading, Immediate Task Performance as well as Memory Performance in Experiment 3*

|  | **Cognitive Offloading** Openings of the Model Window | | No analyses with forced condition due to no variance in offloading data | |
| --- | --- | --- | --- | --- |
|  | Choice | |  |  |
|  | Uninformed | Informed |  |  |
| **Working Memory Capacity**  Visual Patterns Test  Corsi Blocks Task | -.22  -.12 | -.39**  .06 |  |  |
|  | Initially Correctly Copied Items | |  |  |
|  | Choice | |  |  |
|  | Uninformed | Informed |  |  |
| Visual Patterns Test  Corsi Blocks Task | -.03  -.002 | .04  -.23 |  |  |
|  | **Immediate Task Performance**  Trial Duration (sec) | | | |
|  | Choice | | Forced | |
|  | Uninformed | Informed | Uninformed | Informed |
| Visual Patterns Test  Corsi Blocks Task | -.50***  -.31* | -.33*  -.24 | -.19  -.36* | -.11  .06 |
|  | **Memory Performance**  Identity | | | |
|  | Choice | | Forced | |
|  | Uninformed | Informed | Uninformed | Informed |
| Visual Patterns Test  Corsi Blocks Task | -.003  .03 | .18  .04 | .07  .14 | .22  .24 |
|  | Identity-Location Bindings | | | |
|  | Choice | | Forced | |
|  | Uninformed | Informed | Uninformed | Informed |
| Visual Patterns Test  Corsi Blocks Task | .01  .10 | .01  .09 | .16  .13 | .17  .28 |

*Note:* * *p* < .05, ** *p* < .01, *** *p* < .001
